# Supplementary material for: Synthesis of Isomeric 3-Benzazecines Decorated with Endocyclic Allene Moiety and Exocyclic Conjugated Double Bond and Evaluation of Their Anticholinesterase Activity
Source: Molecules. 2022 Sep 23;27(19):6276. doi: 10.3390/molecules27196276 (PMC9571408; doi:10.3390/molecules27196276)
Supplement: Supplementary file 1 [file molecules-27-06276-s001.zip › molecules-1901928-supplementary.pdf]

Supporting Information

Synthesis of isomeric 3-benzazecines decorated with endocyclic allene moiety and exocyclic conjugated double bond and evaluation of their anti-cholinesterase activity

Alexander A. Titov [1,¶], Rosa Purgatorio [2,¶], Arina Y. Obydennik [1], Anna V. Listratova [1], Tatiana N. Borisova [1], Modesto de Candia [2], Marco Catto [2], Cosimo D. Altomare [2,*], Alexey V. Varlamov [1] and Leonid G. Voskressensky [1]

[1] Organic Chemistry Department, Peoples' Friendship University of Russia (RUDN University), 6 Miklukho-Maklaya St, Moscow, 117198, Russian Federation

[2] Department of Pharmacy-Pharmaceutical Sciences, University of Bari Aldo Moro, Via E. Orabona 4, 70125 Bari, Italy;

* Correspondence: cosimodamiano.altomare@uniba.it; Tel.: +39-080-5442781
¶ These authors equally contributed

Table of content

Copies of ^{1}H and ^{13}C NMR spectra..2

Copies of ^{1}H and ^{13}C NMR spectra

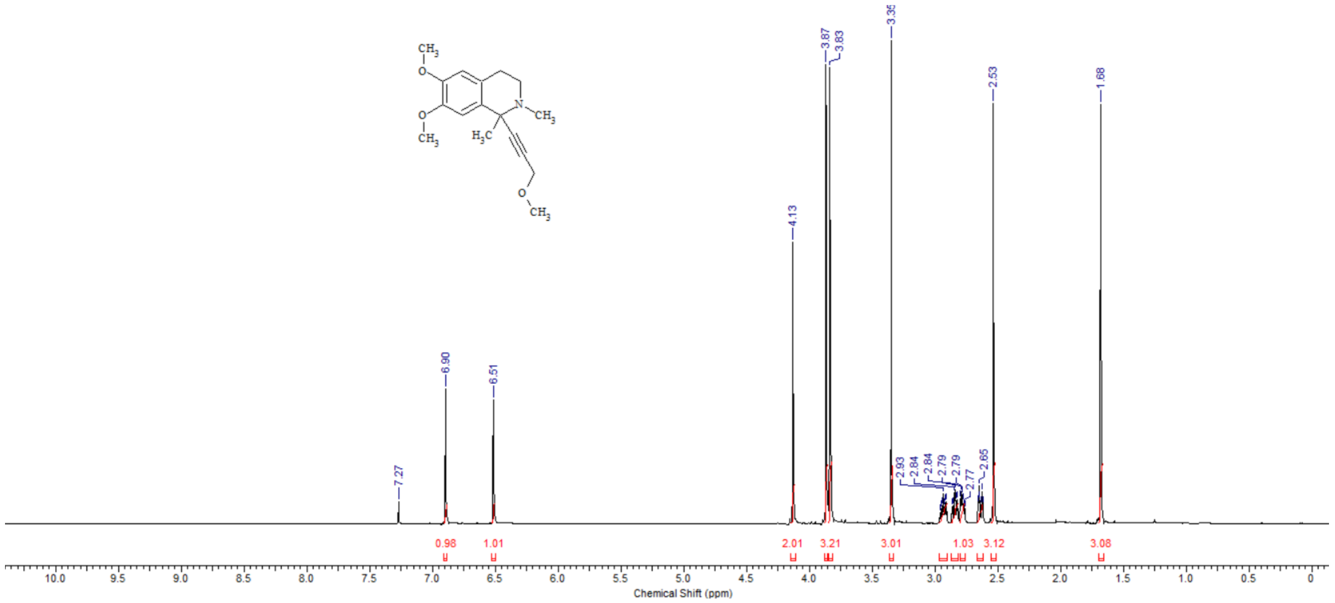

Figure S1: ^{1}H NMR spectrum of compound **2a**

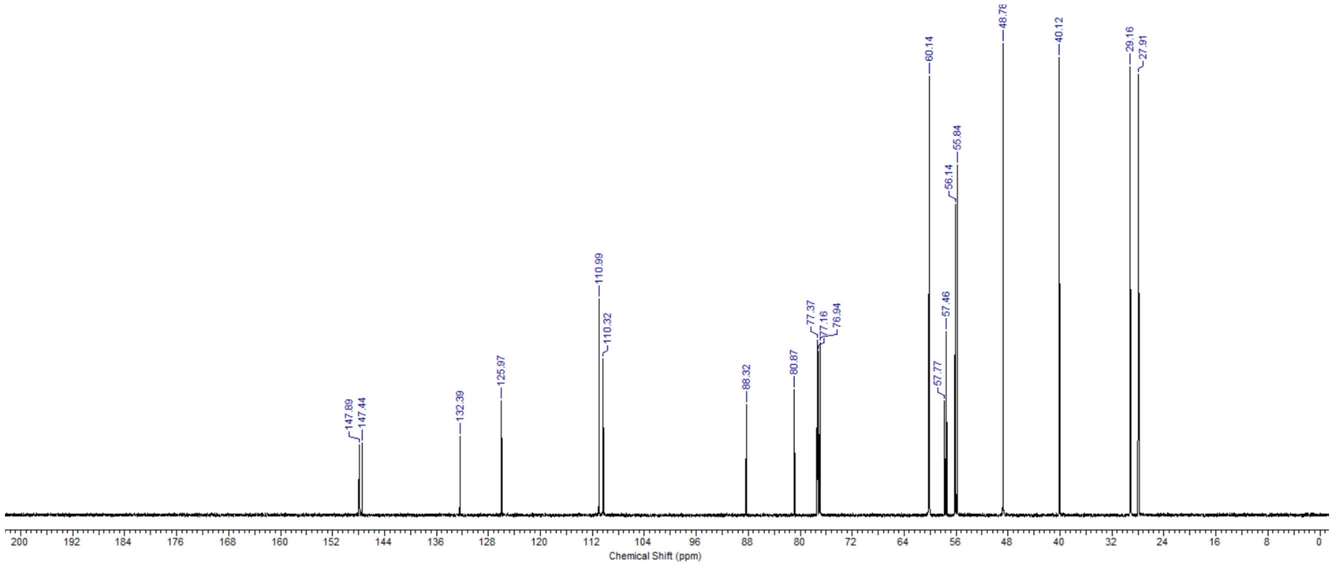

Figure S2: ^{13}C NMR spectrum of compound **2a**

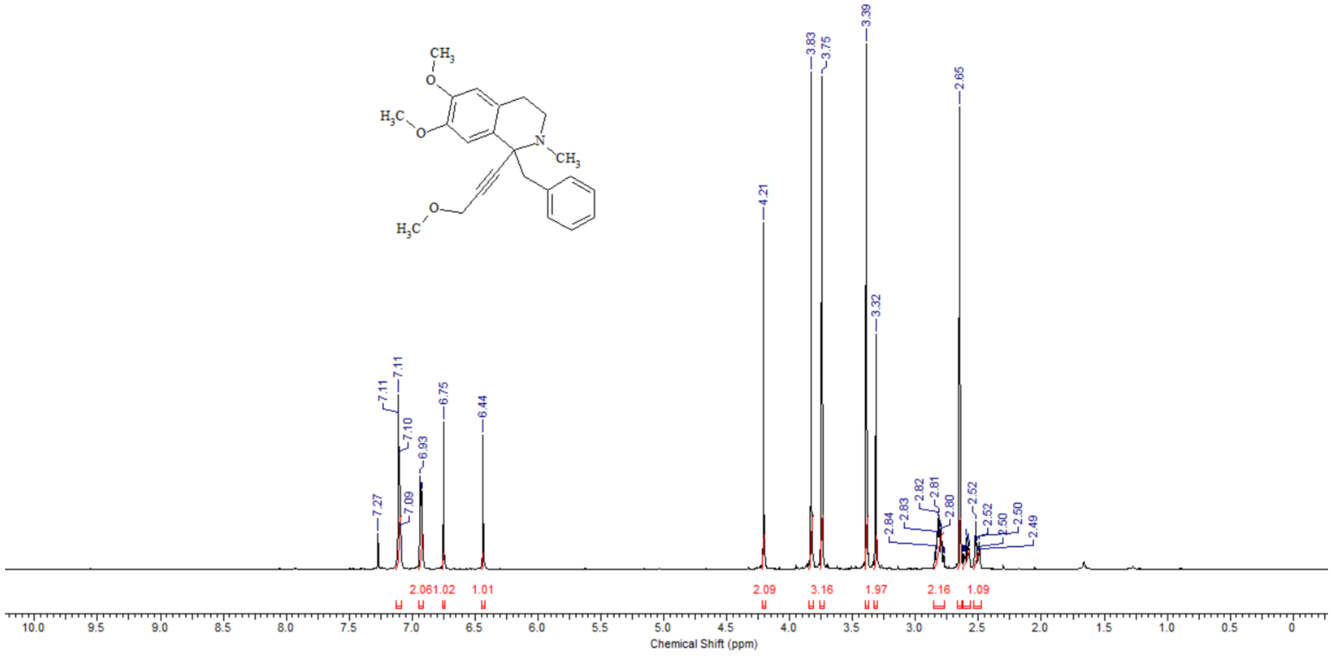

Figure S3: ^{1}H NMR spectrum of compound **2b**

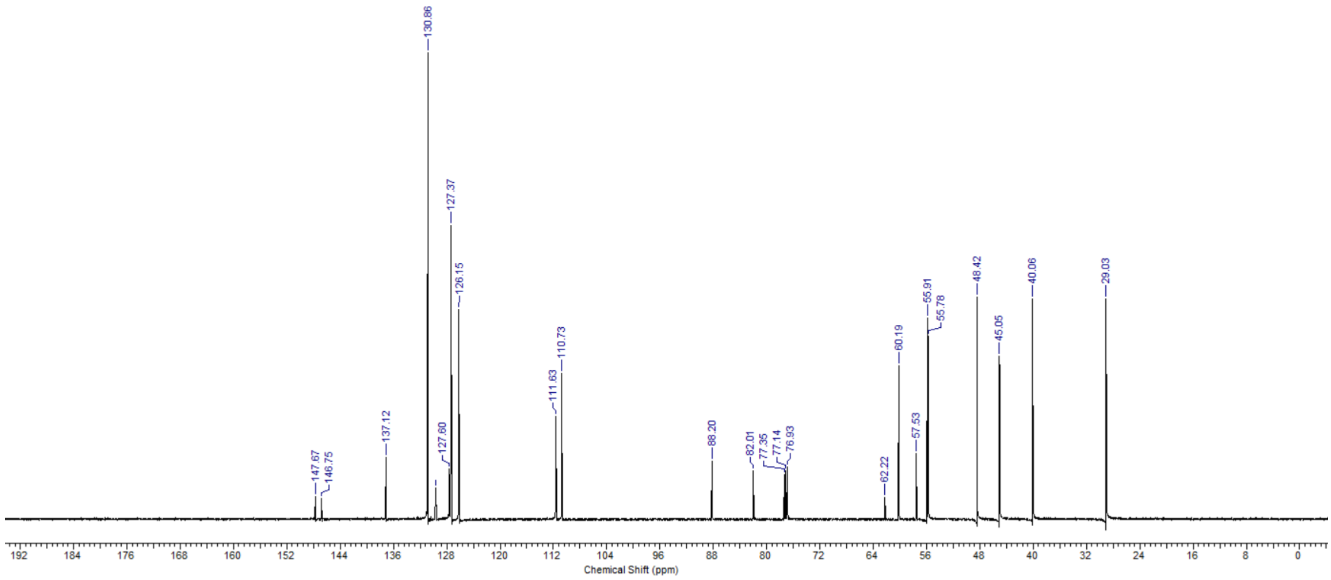

Figure S4: ^{13}C NMR spectrum of compound **2b**

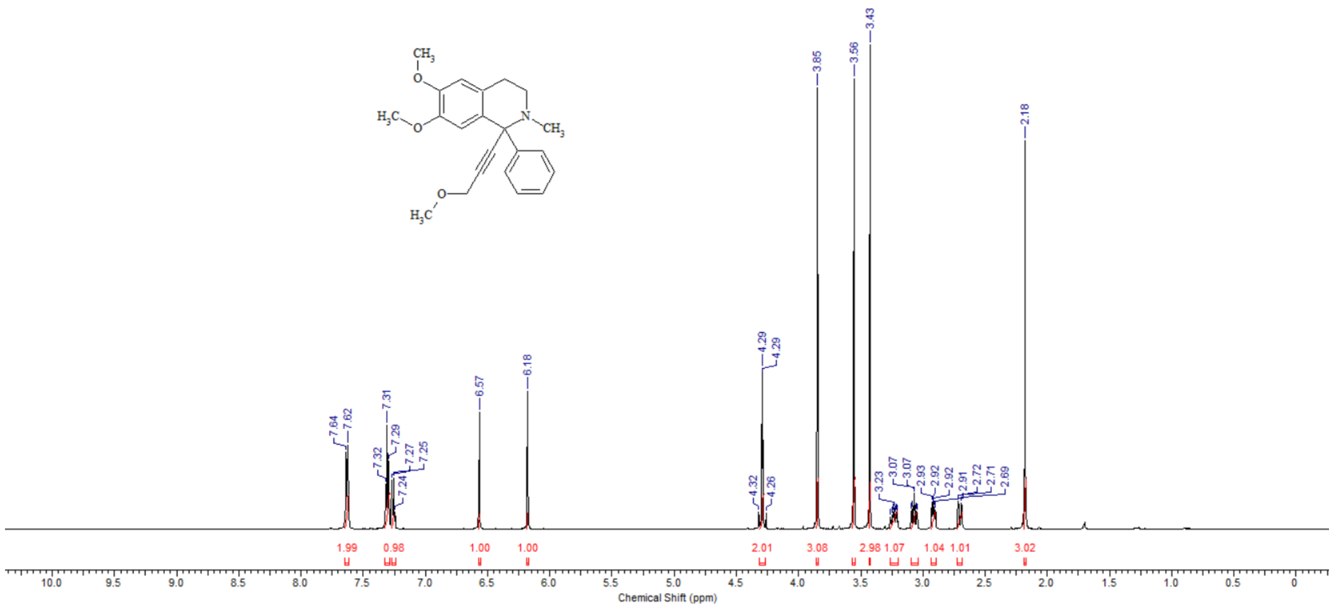

Figure S5: ^{1}H NMR spectrum of compound **2c**

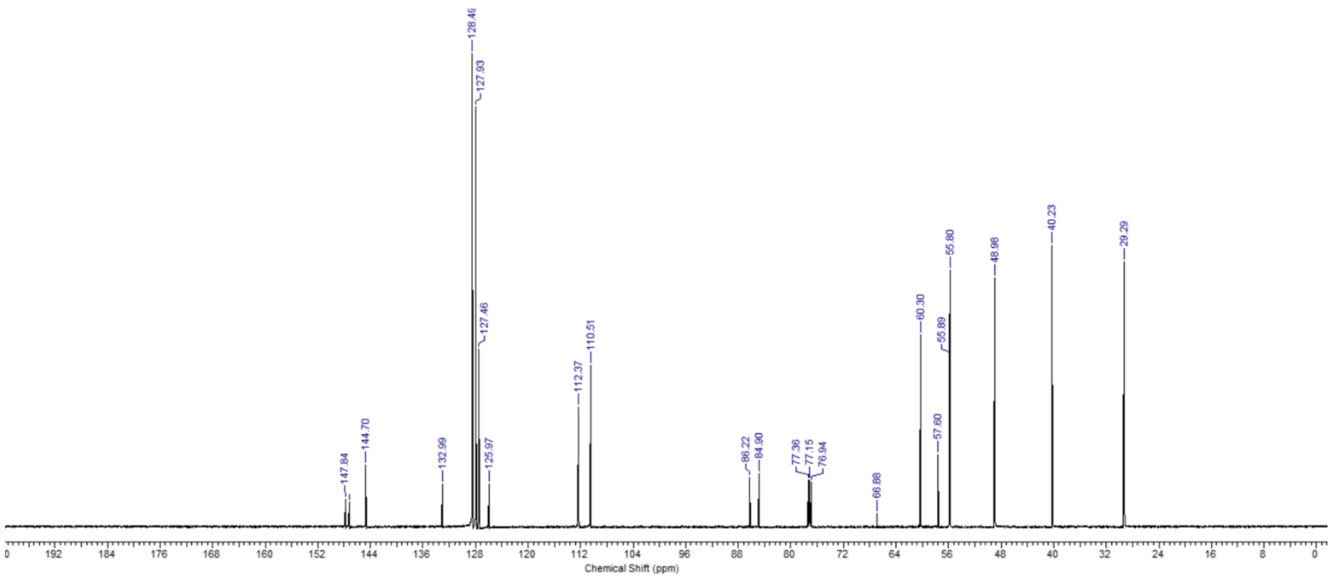

Figure S6: ^{13}C NMR spectrum of compound **2c**

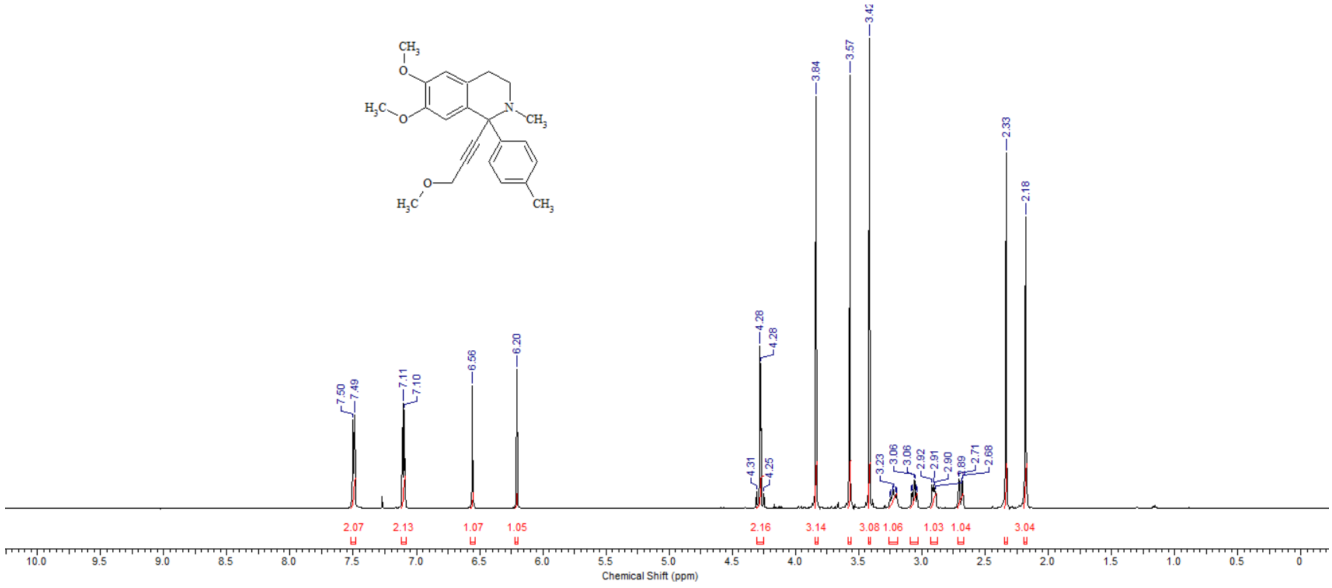

Figure S7: ^{1}H NMR spectrum of compound **2d**

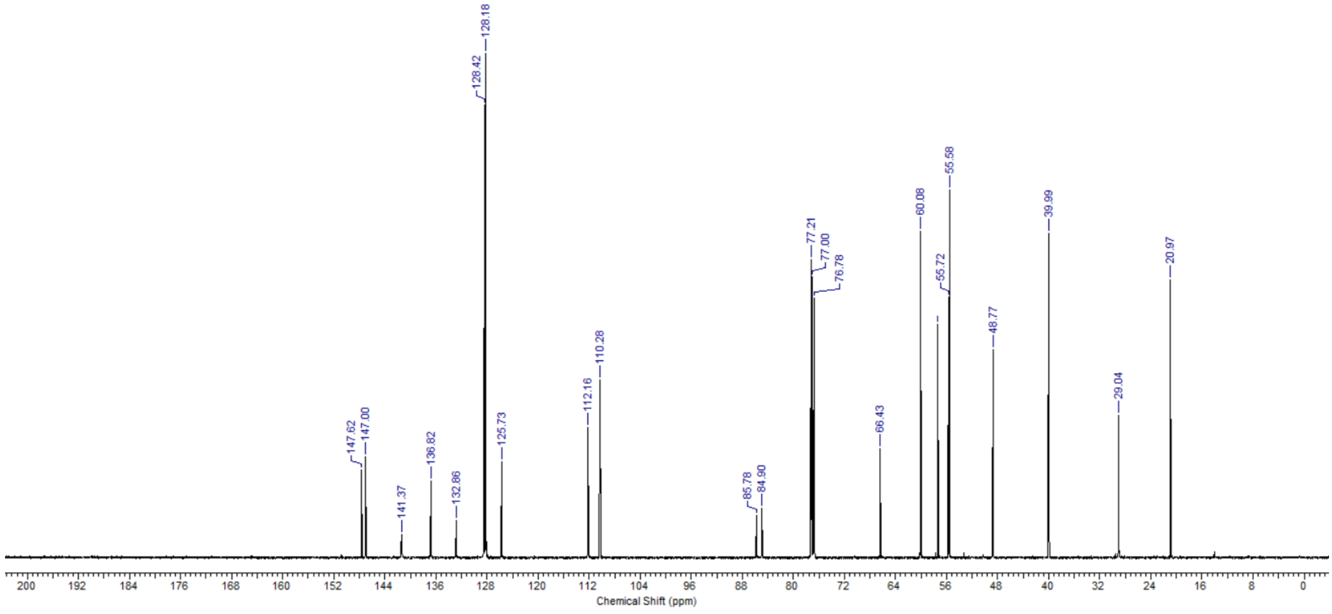

Figure S8: ^{13}C NMR spectrum of compound **2d**

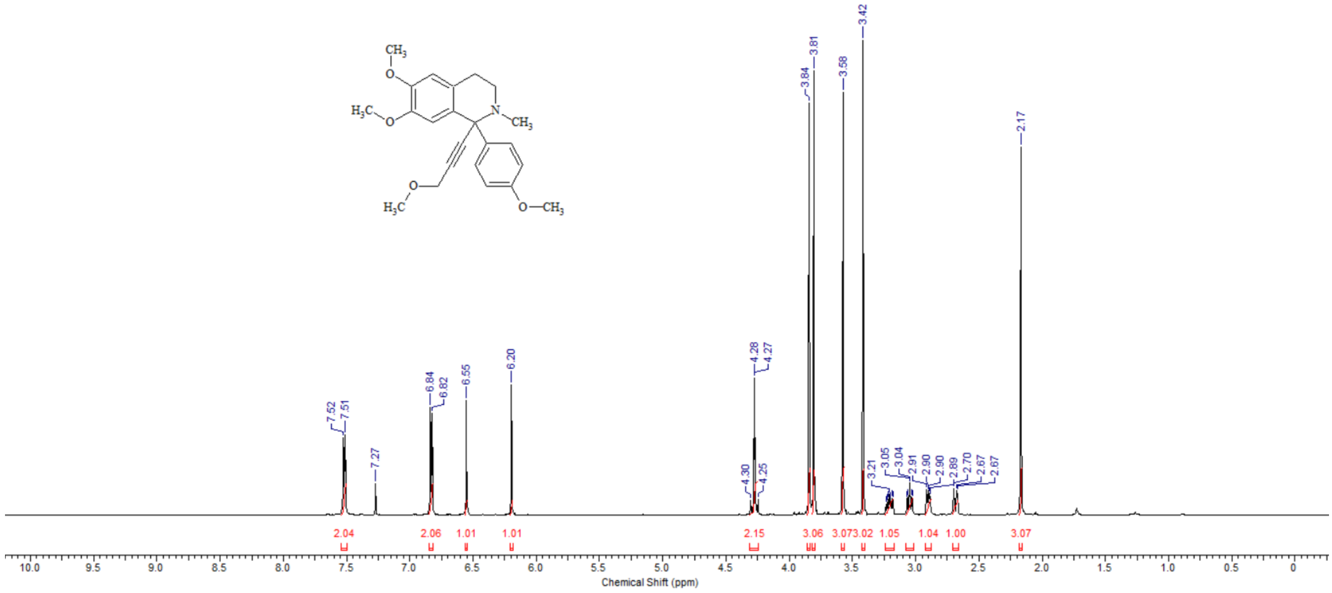

Figure S9: ^{1}H NMR spectrum of compound **2e**

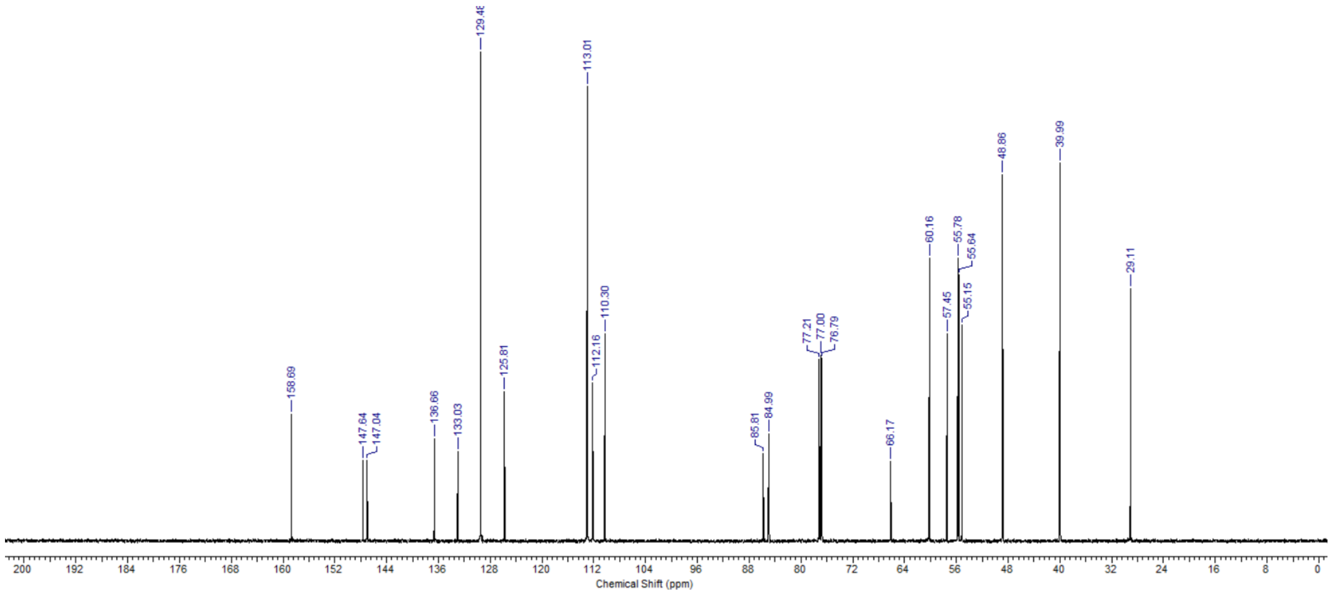

Figure S10: ^{13}C NMR spectrum of compound **2e**

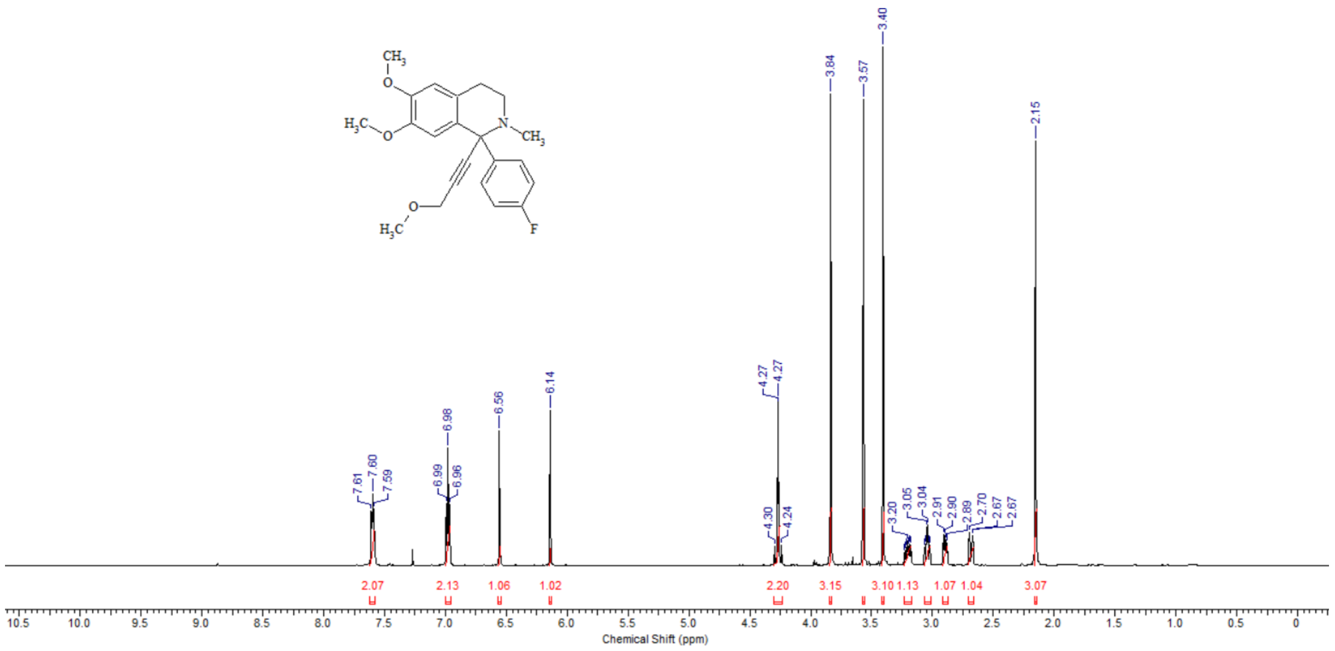

Figure S11: ^{1}H NMR spectrum of compound **2f**

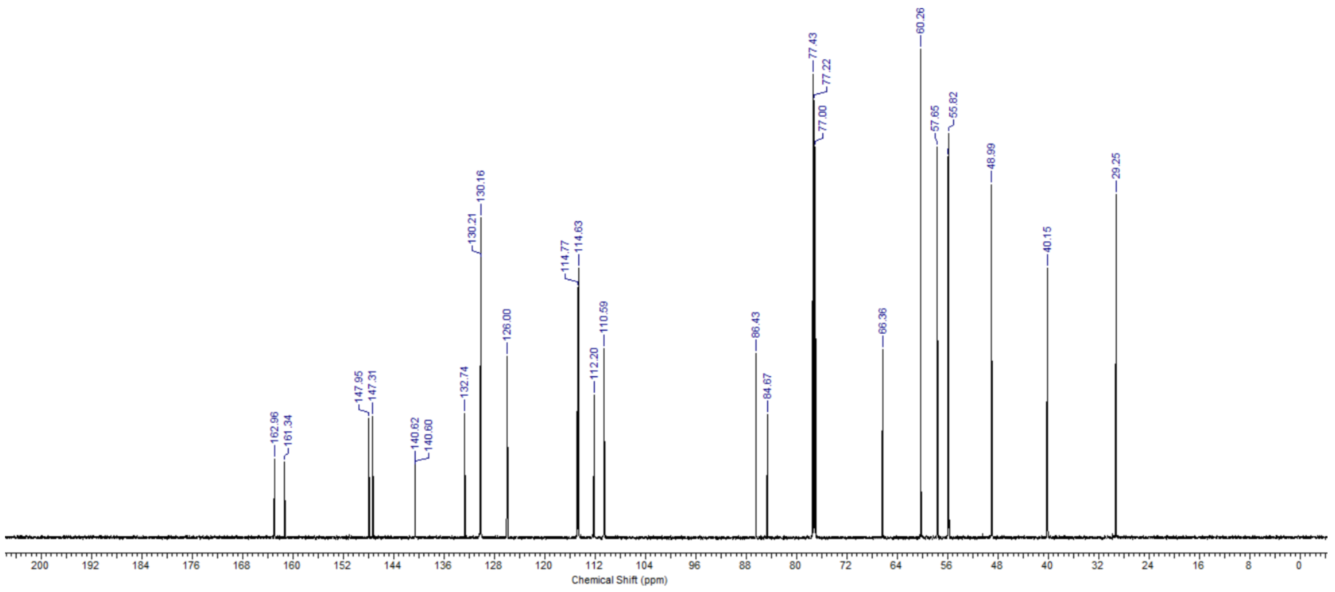

Figure S12: ^{13}C NMR spectrum of compound **2f**

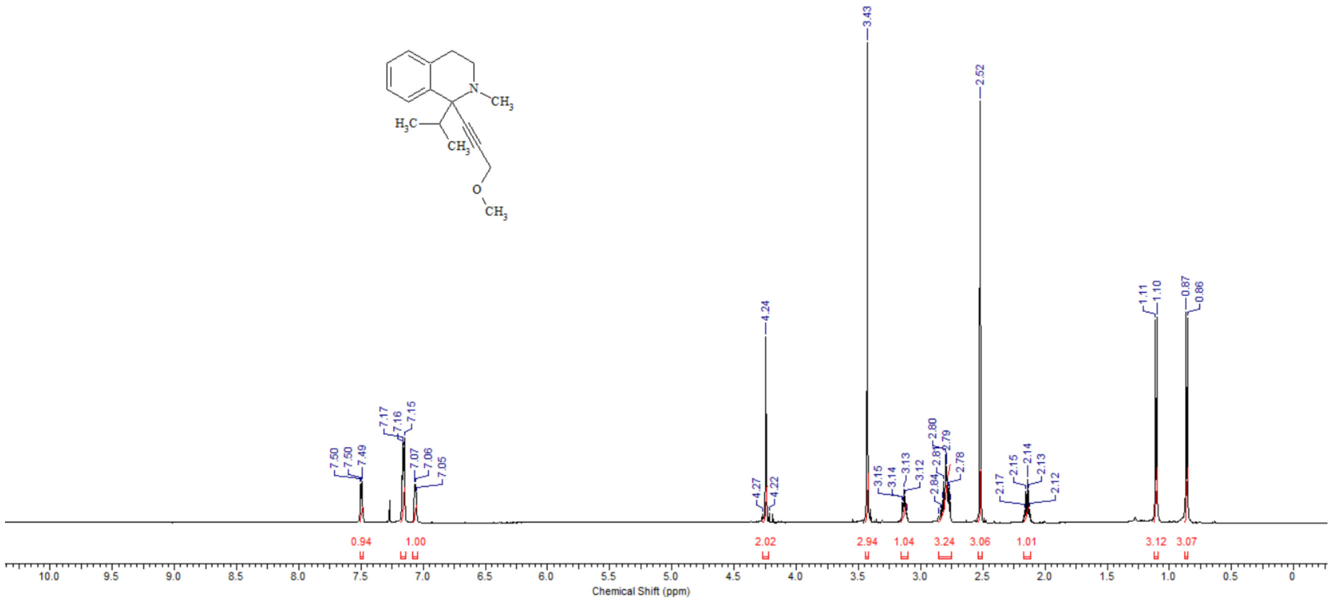

Figure S13: ^{1}H NMR spectrum of compound **2g**

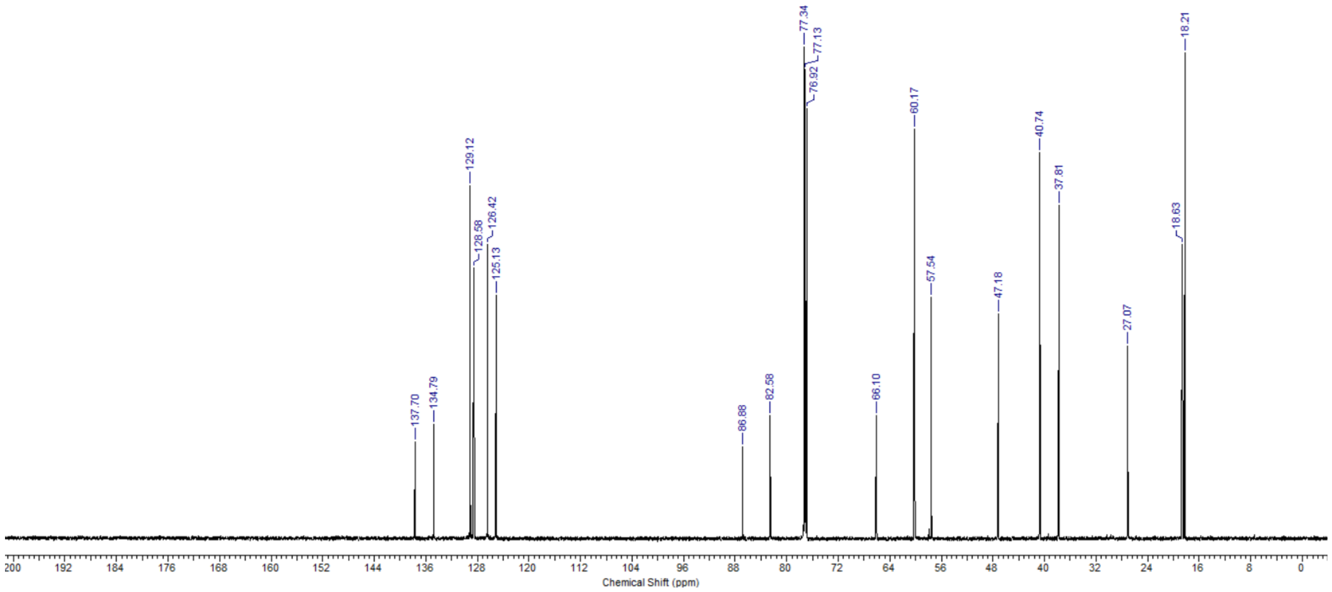

Figure S14: ^{13}C NMR spectrum of compound **2g**

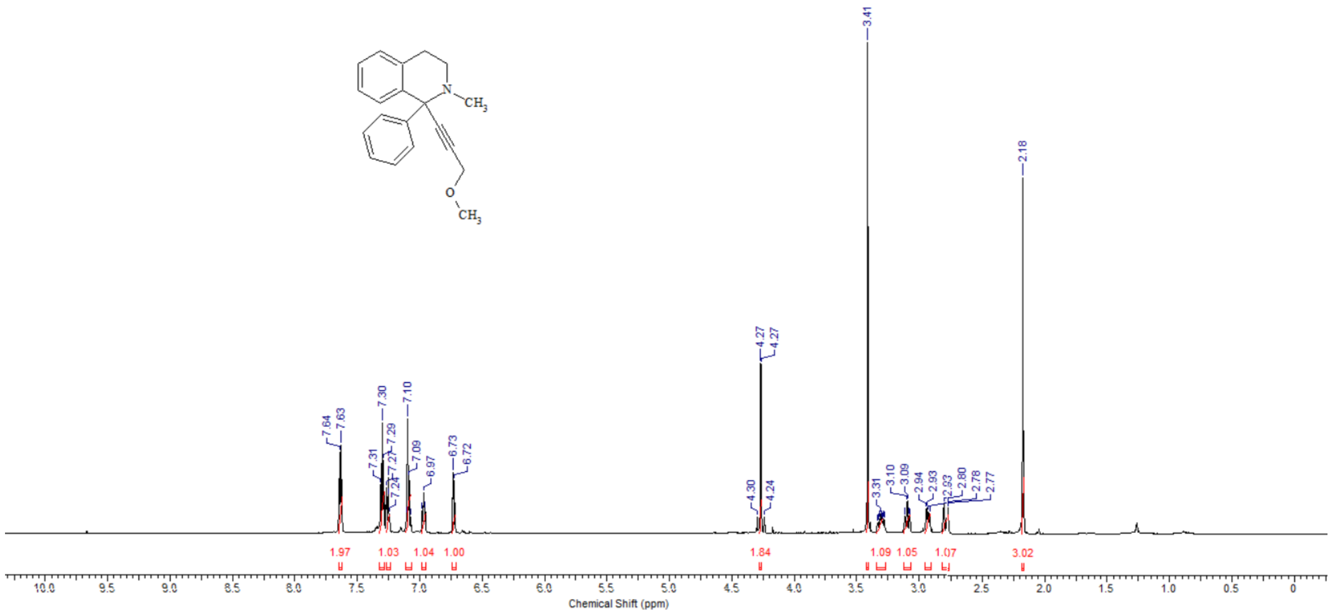

Figure S15: ^{1}H NMR spectrum of compound **2h**

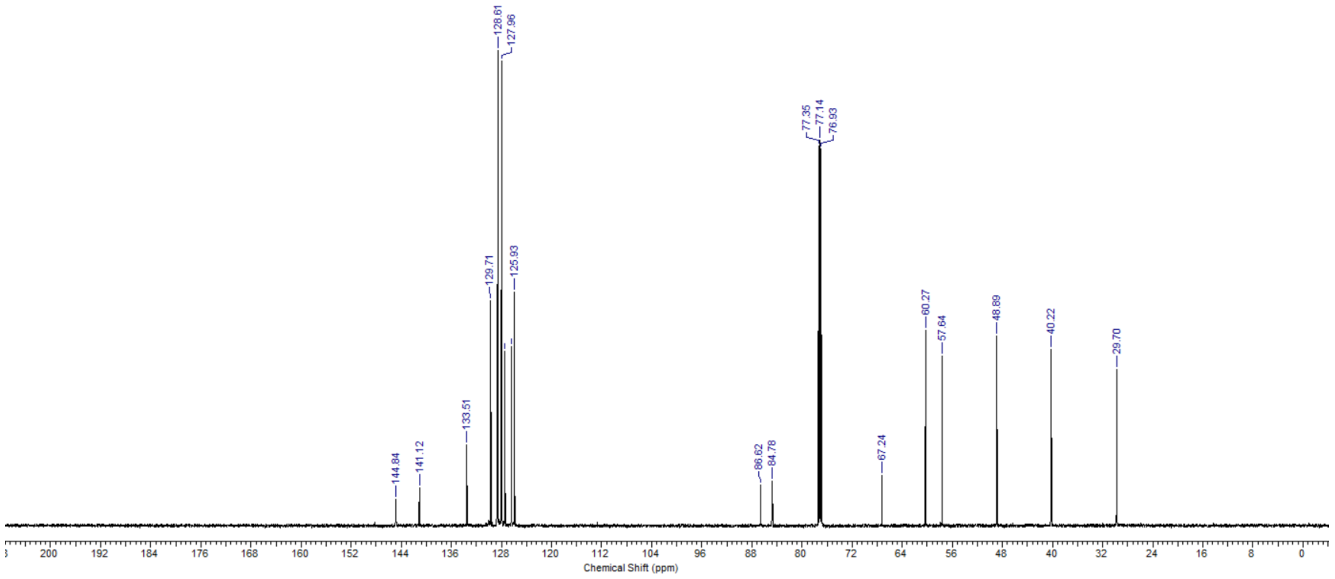

Figure S16: ^{13}C NMR spectrum of compound **2h**

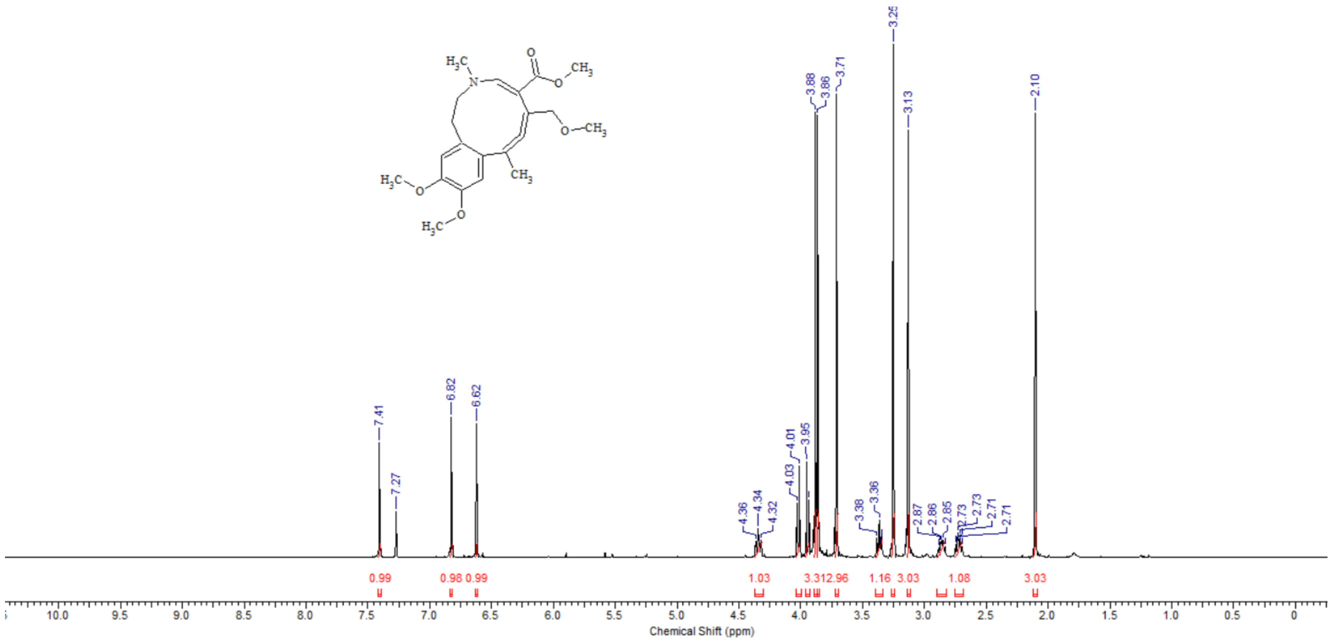

Figure S17: ^{1}H NMR spectrum of compound **3a**

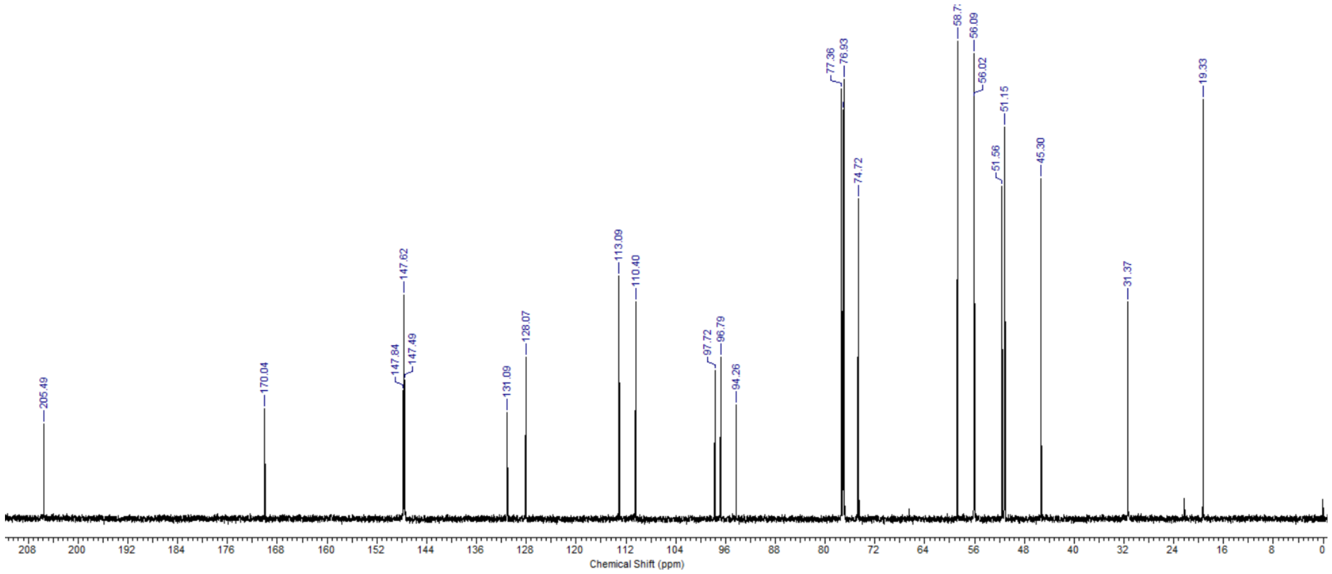

Figure S18: ^{13}C NMR spectrum of compound **3a**

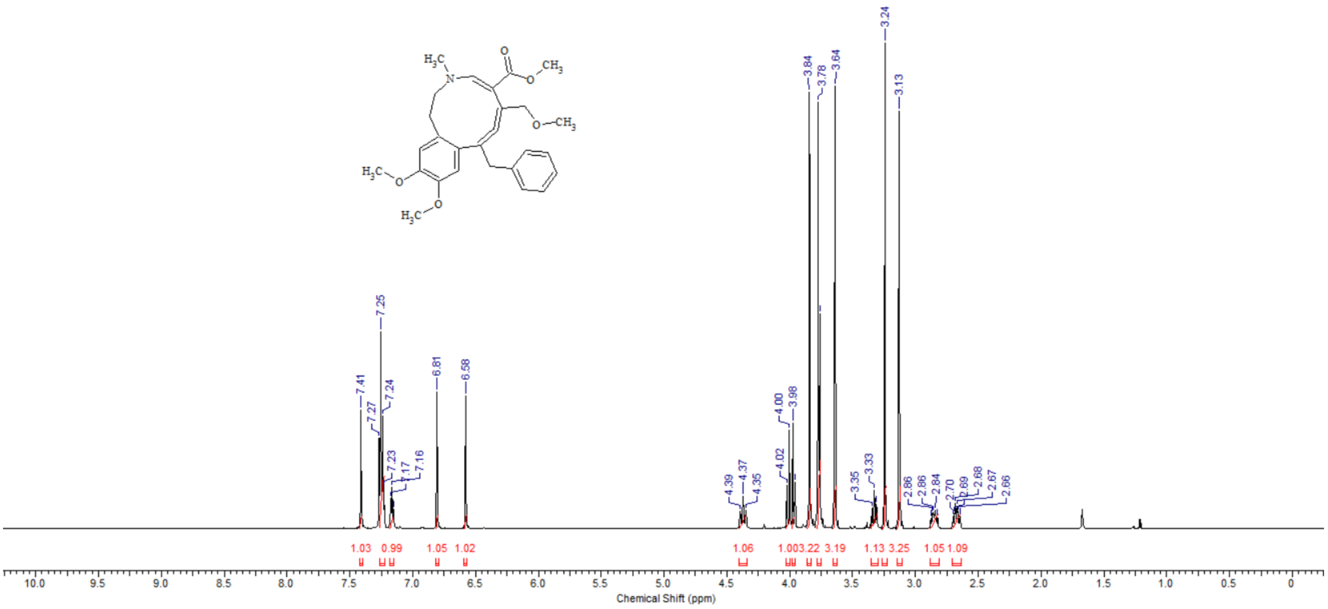

Figure S19: ^{1}H NMR spectrum of compound **3b**

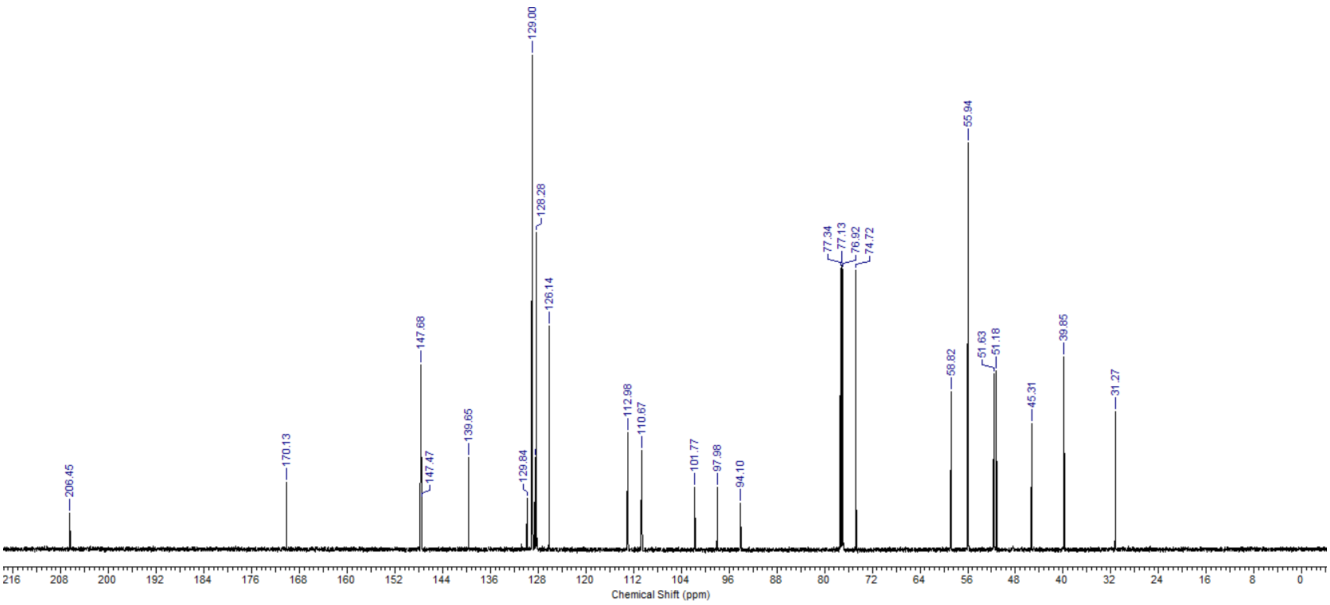

Figure S20: ^{13}C NMR spectrum of compound **3b**

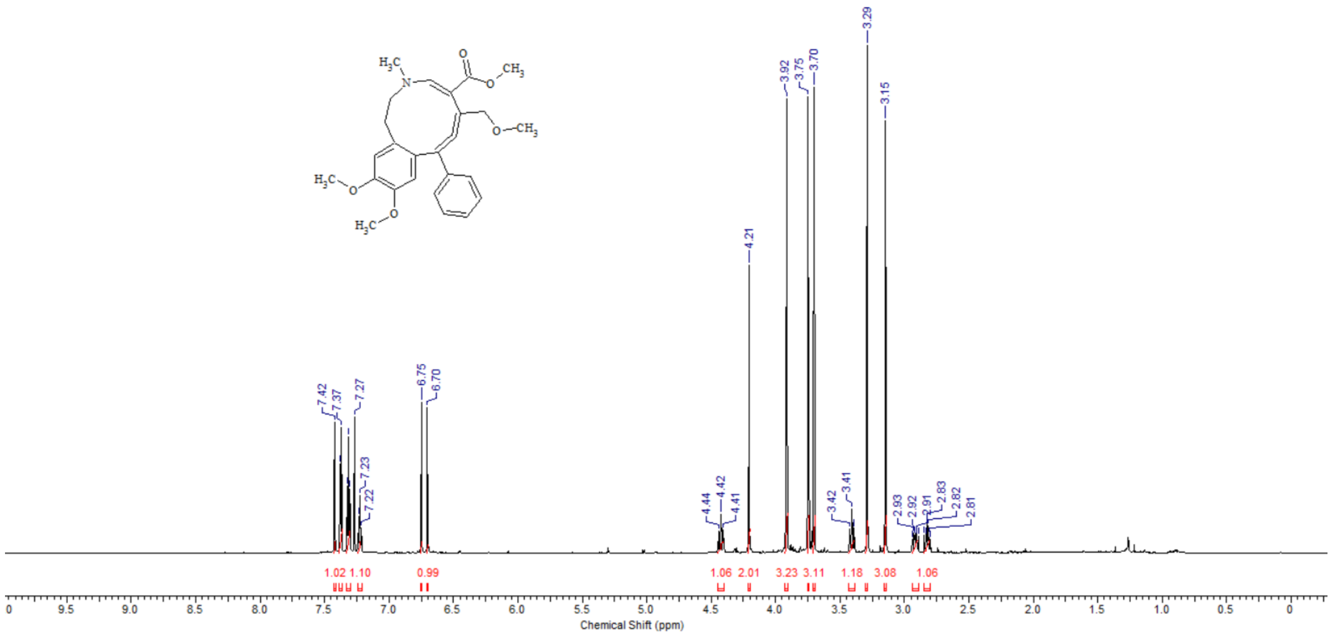

Figure S21: ^{1}H NMR spectrum of compound **3c**

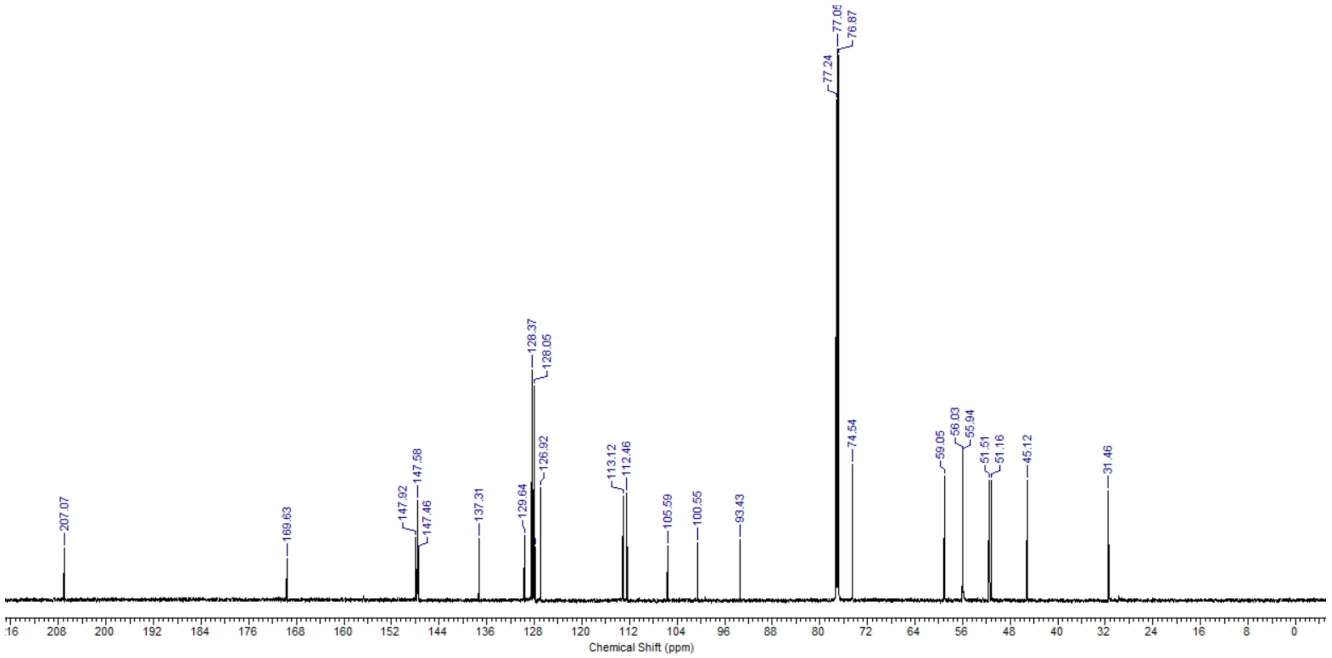

Figure S22: ^{13}C NMR spectrum of compound **3c**

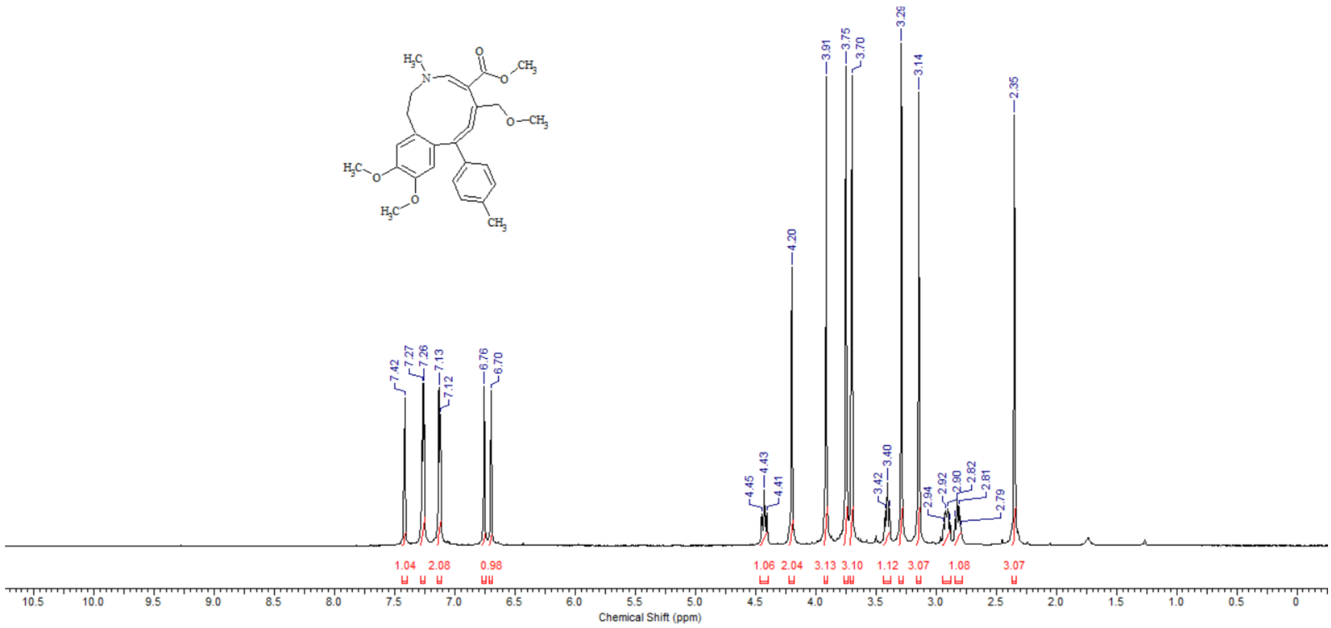

Figure S23: ^{1}H NMR spectrum of compound **3d**

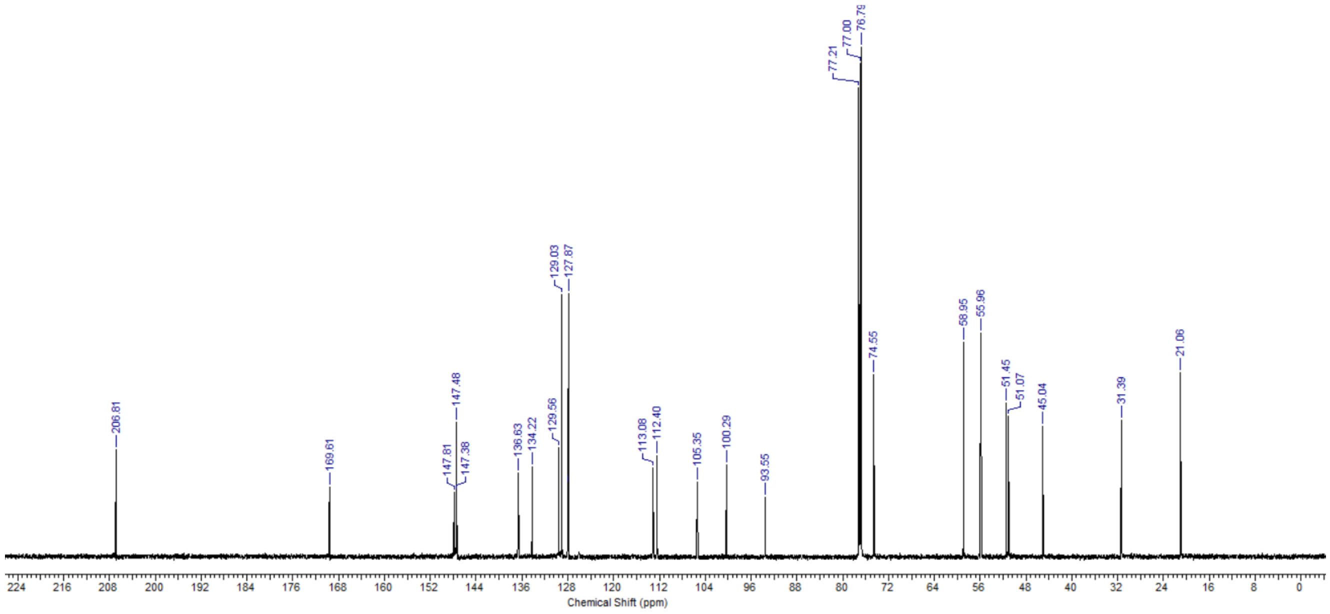

Figure S24: ^{13}C NMR spectrum of compound **3d**

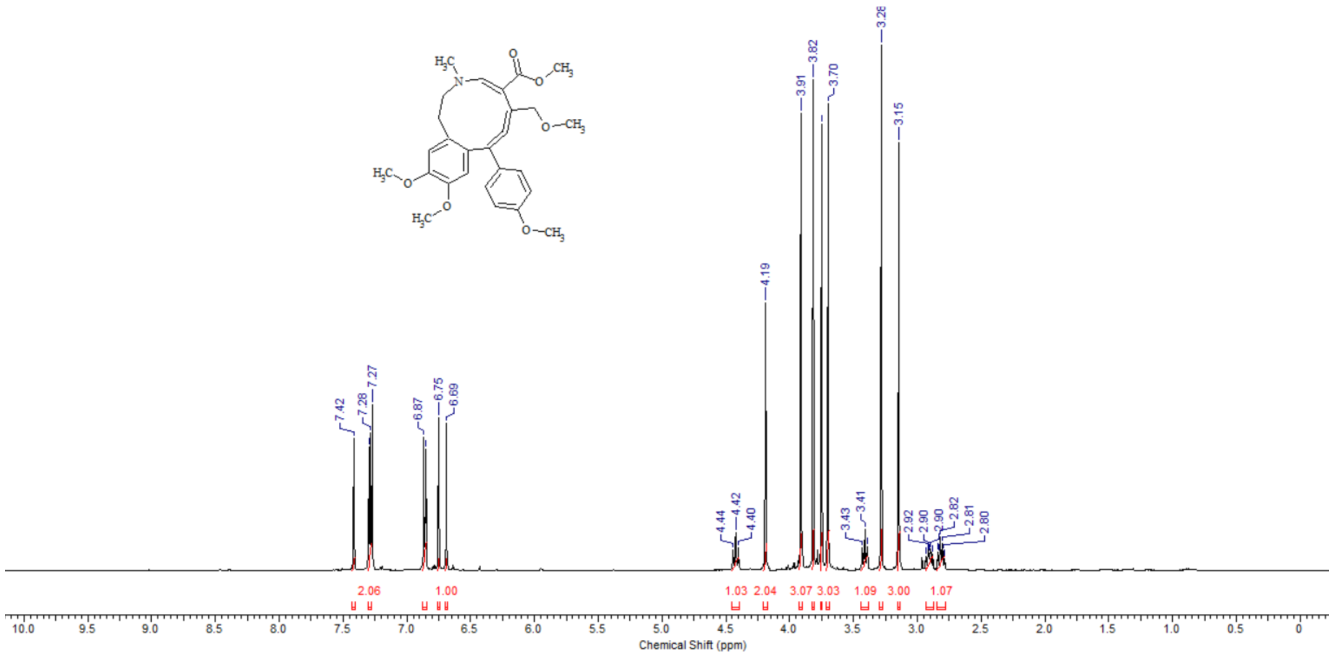

Figure S25: ^{1}H NMR spectrum of compound **3e**

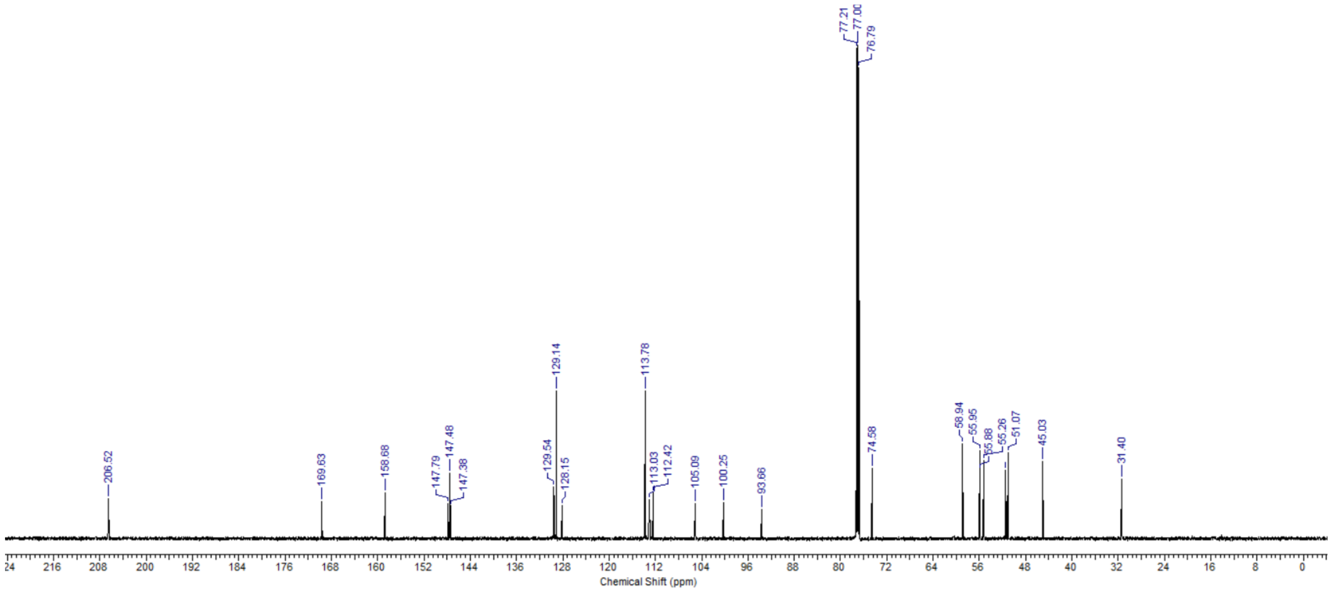

Figure S26: ^{13}C NMR spectrum of compound **3e**

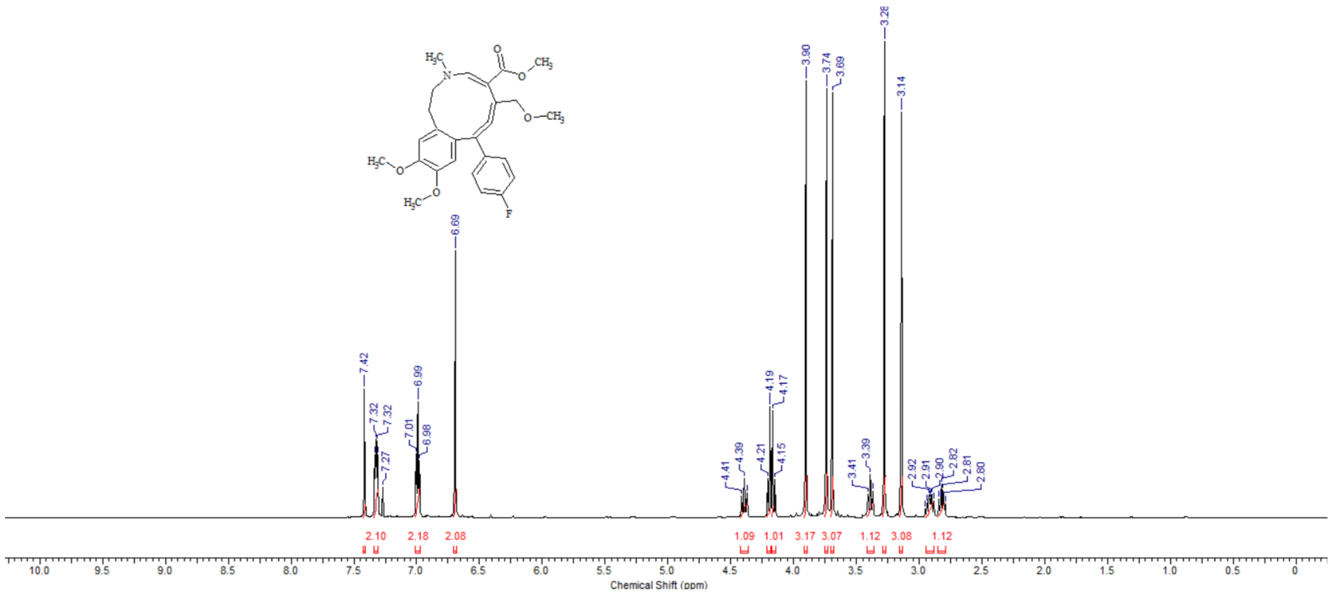

Figure S27: ^{1}H NMR spectrum of compound **3f**

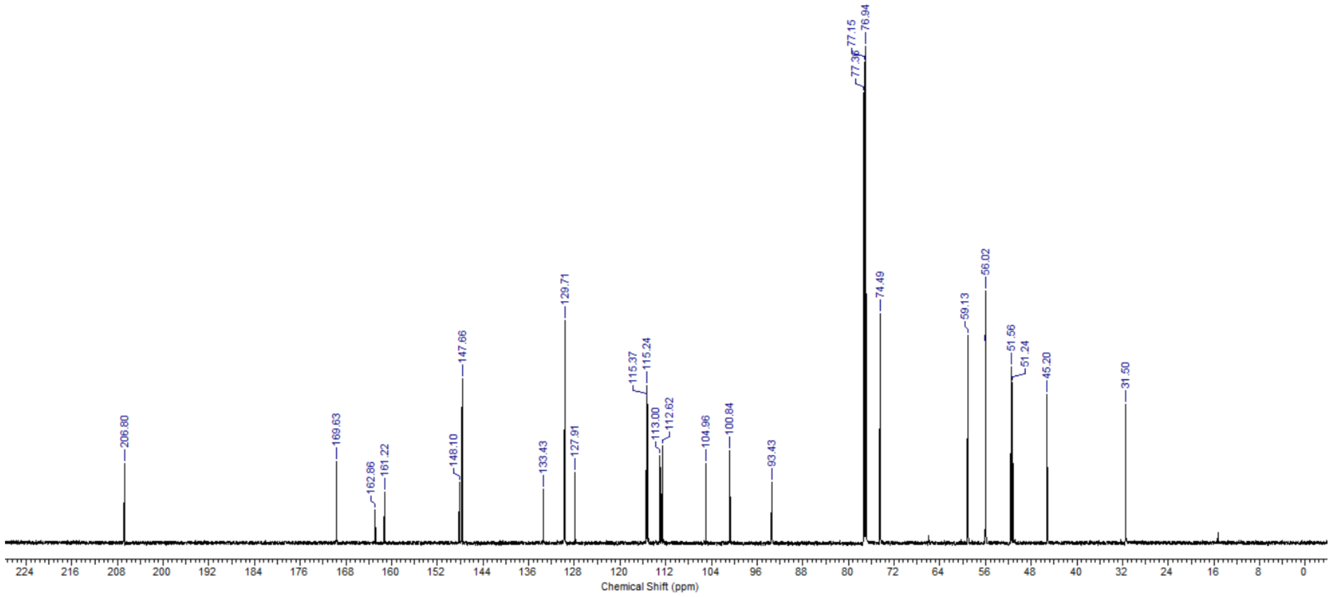

Figure S28: ^{13}C NMR spectrum of compound **3f**

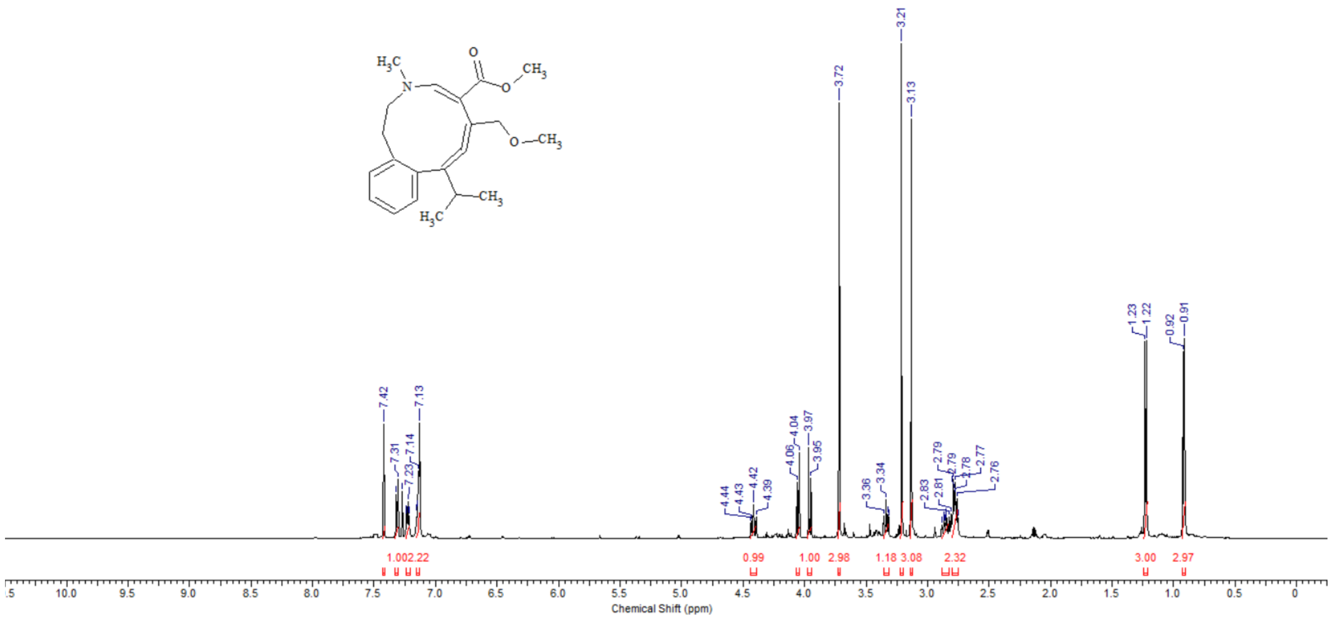

Figure S29: ^{1}H NMR spectrum of compound **3g**

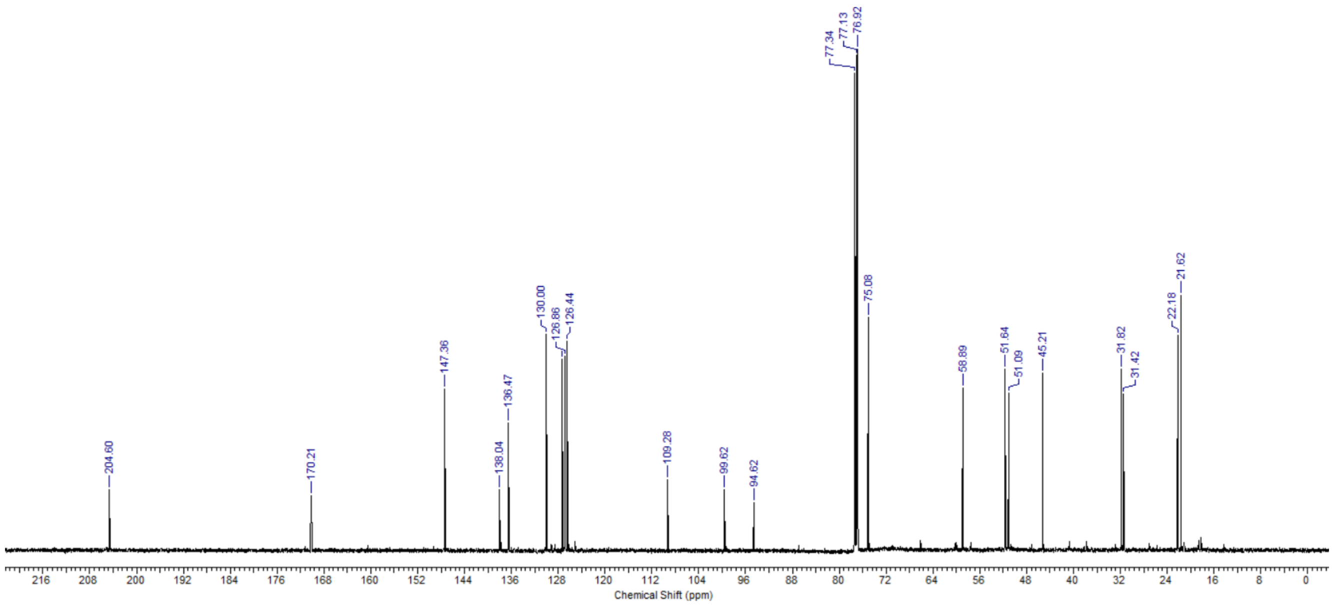

Figure S30: ^{13}C NMR spectrum of compound **3g**

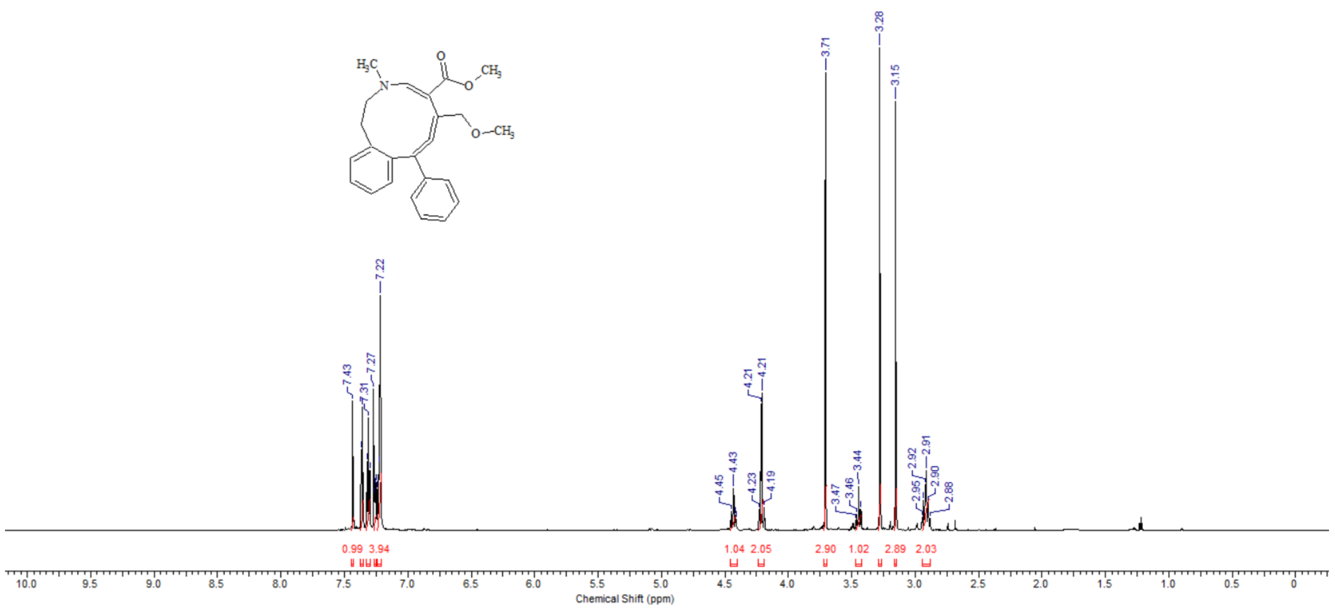

Figure S31: ^{1}H NMR spectrum of compound **3h**

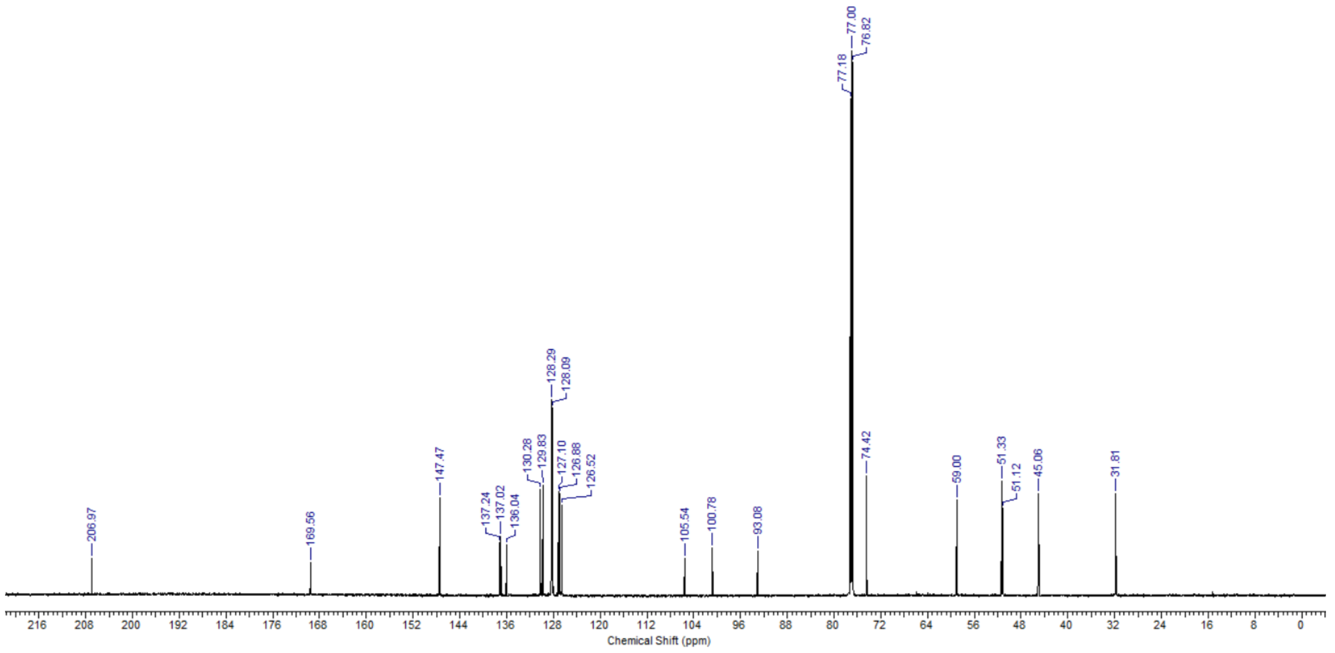

Figure S32: ^{13}C NMR spectrum of compound **3h**

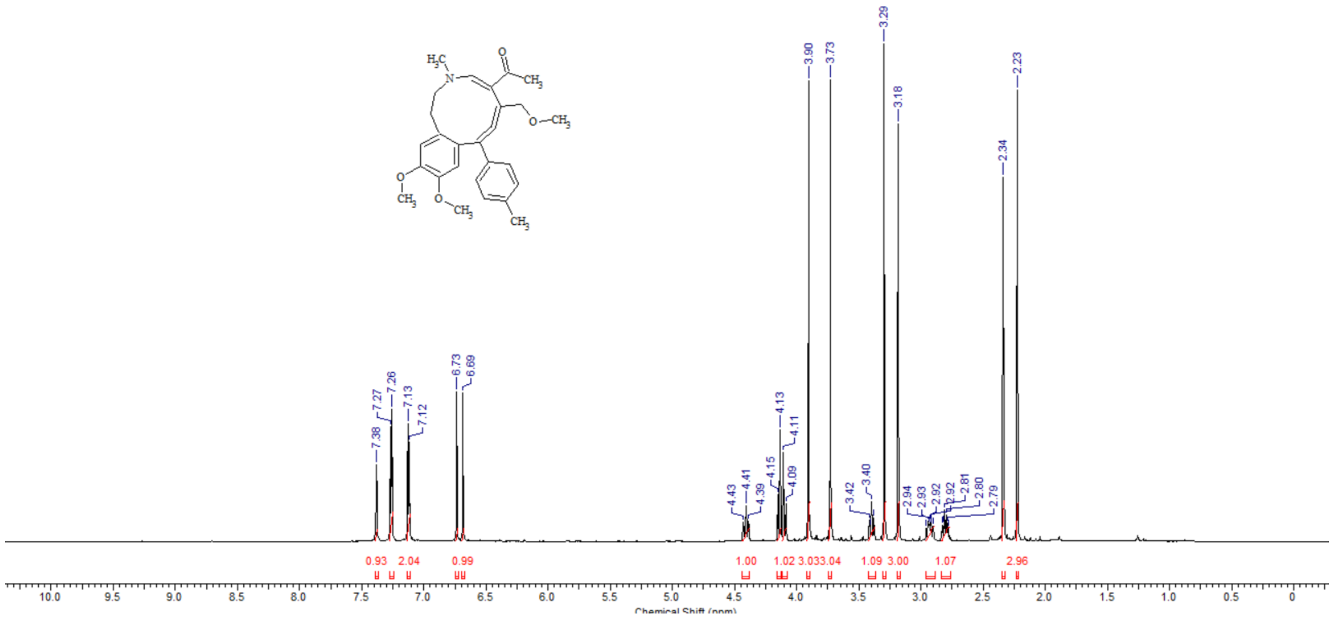

Figure S33: ^{1}H NMR spectrum of compound **3i**

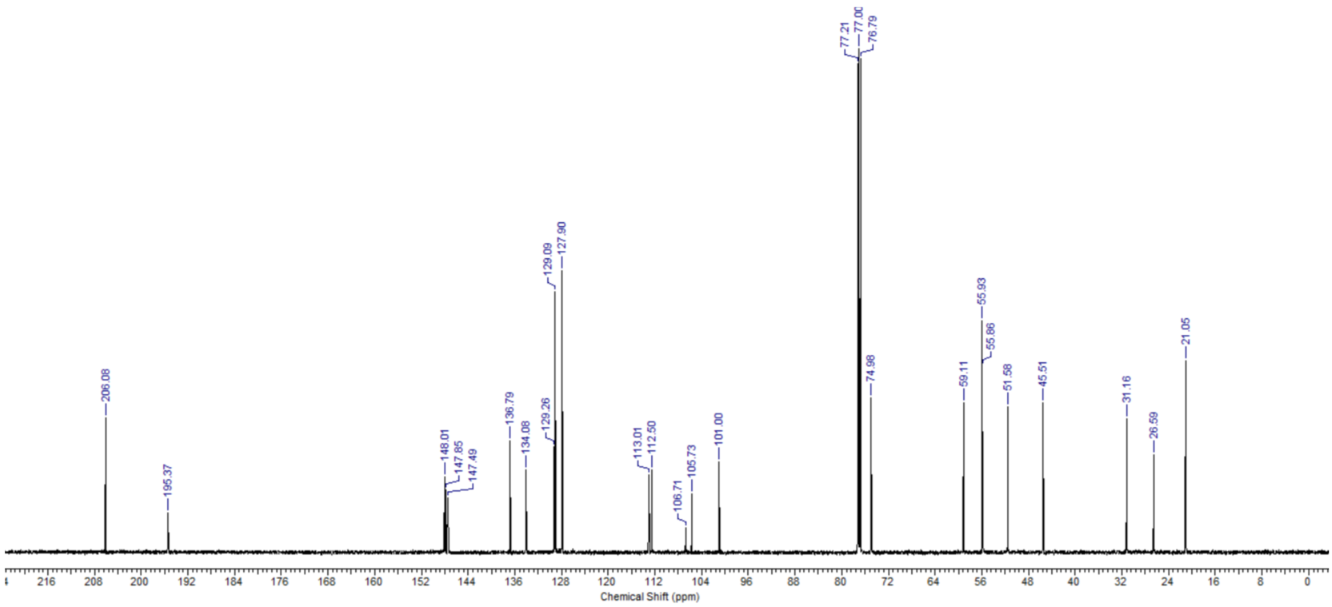

Figure S34: ^{13}C NMR spectrum of compound **3i**

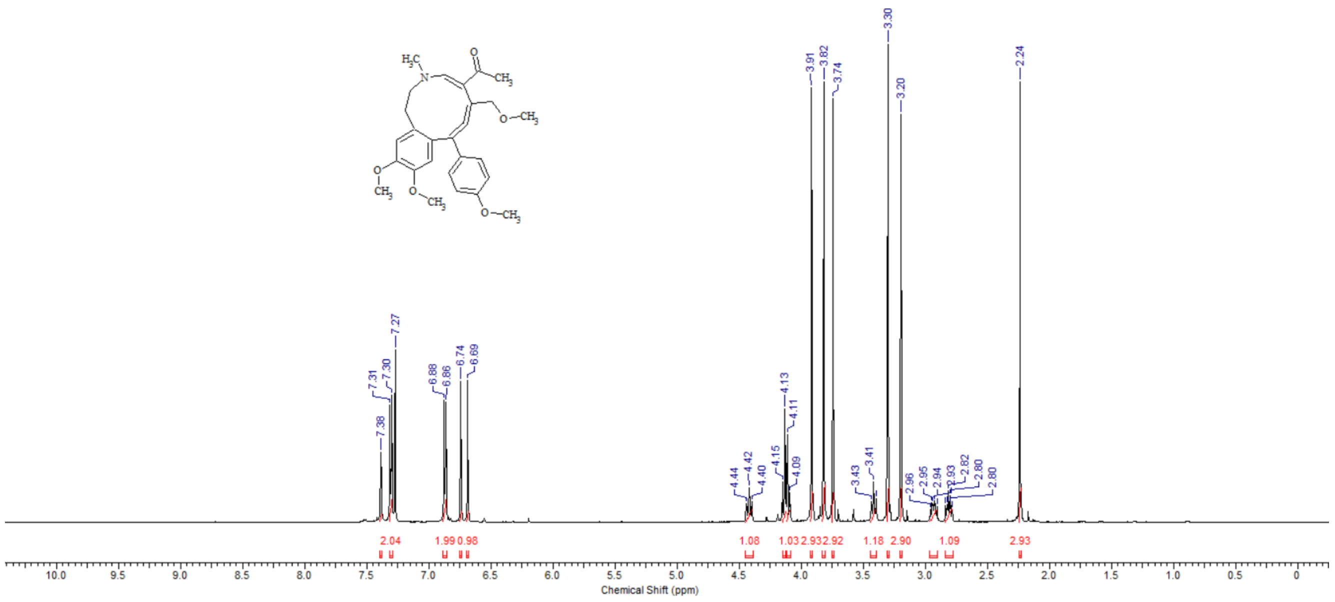

Figure S35: ^{1}H NMR spectrum of compound **3j**

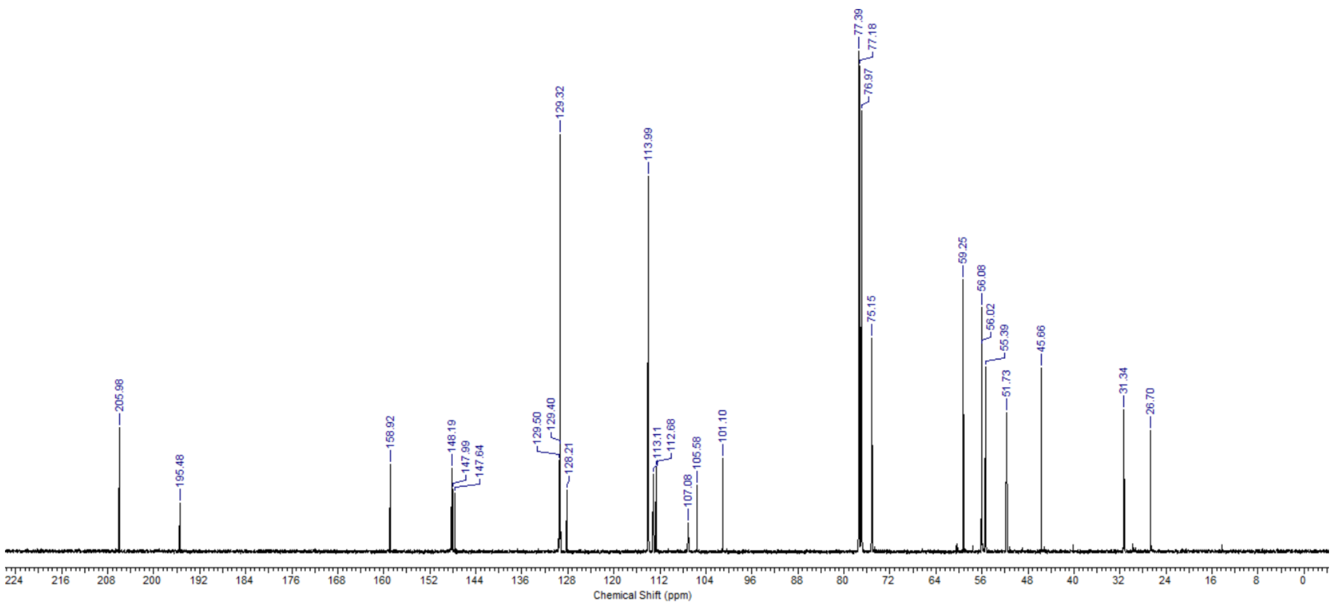

Figure S36: ^{13}C NMR spectrum of compound **3j**

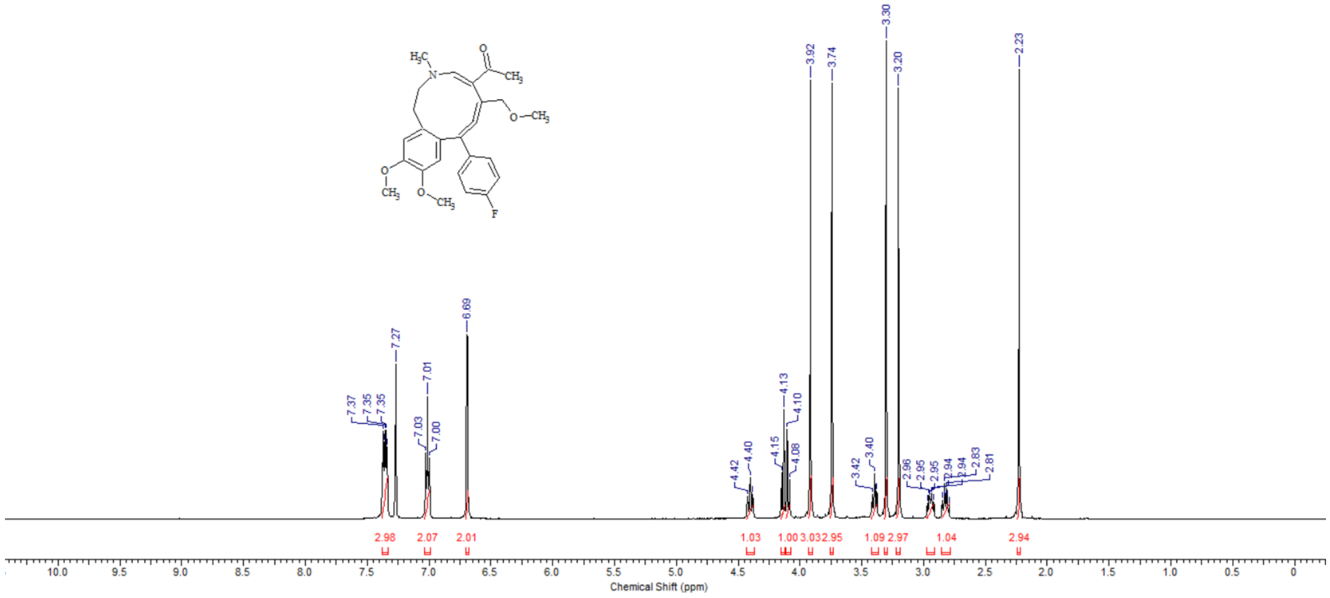

Figure S37: ^{1}H NMR spectrum of compound **3k**

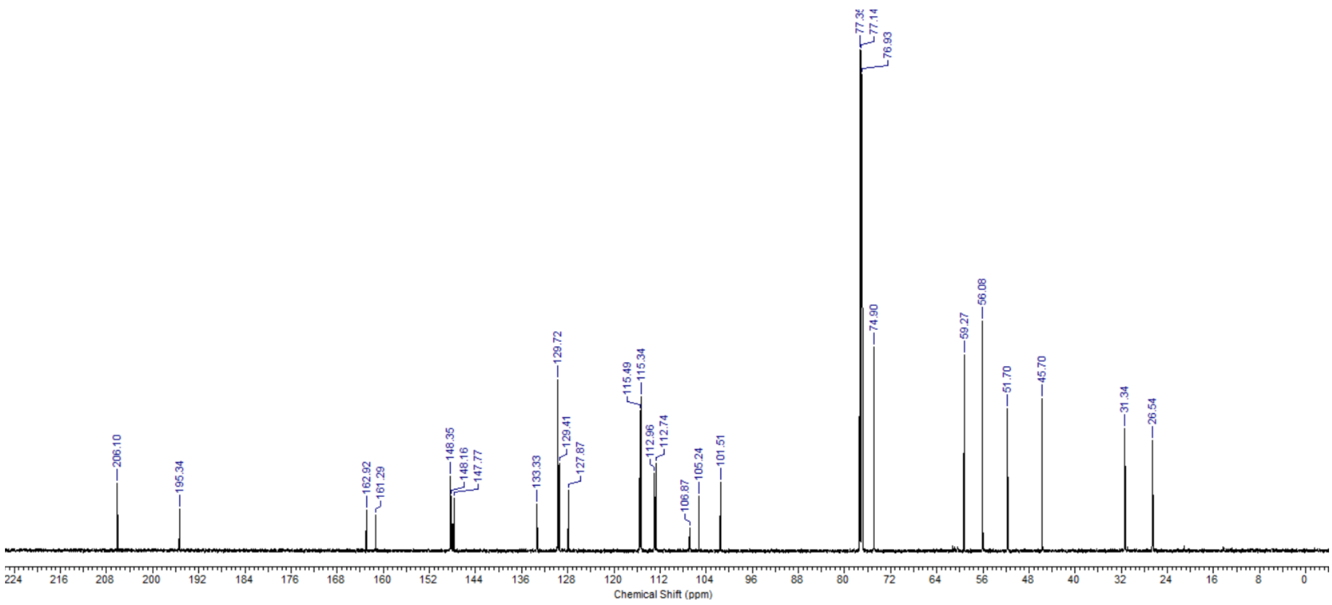

Figure S38: ^{13}C NMR spectrum of compound **3k**

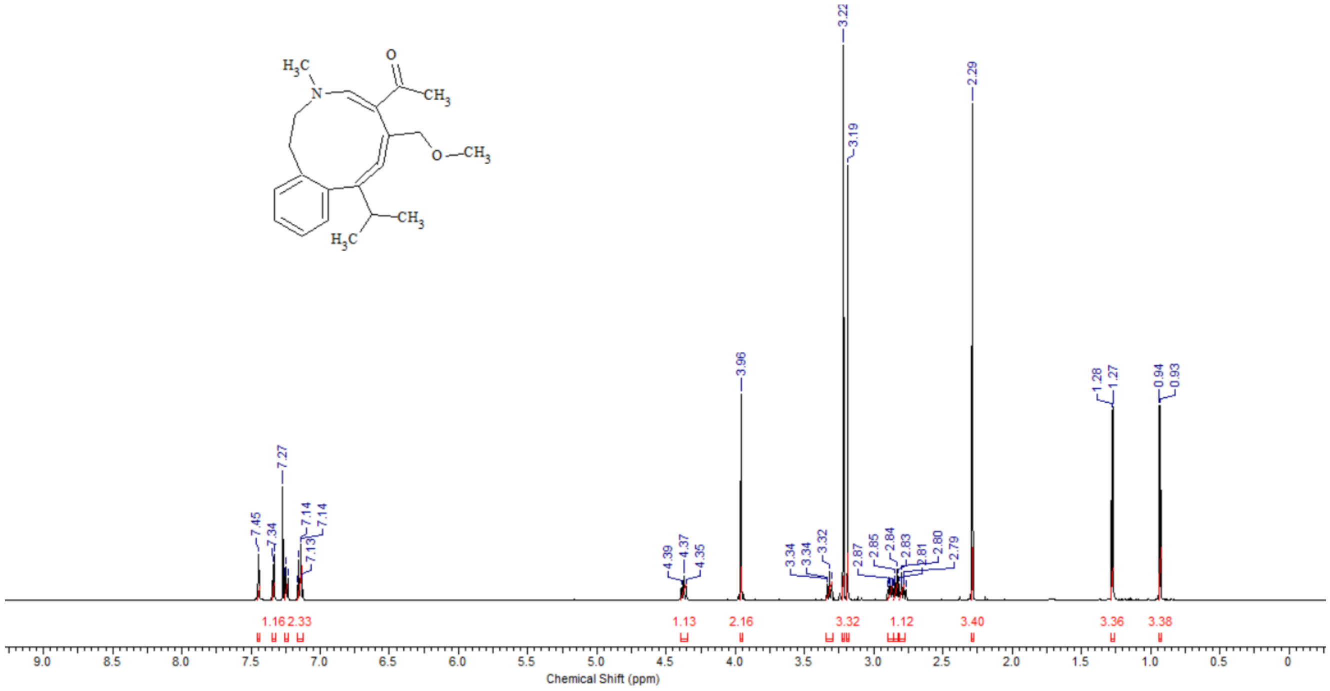

Figure S39: ^{1}H NMR spectrum of compound **3l**

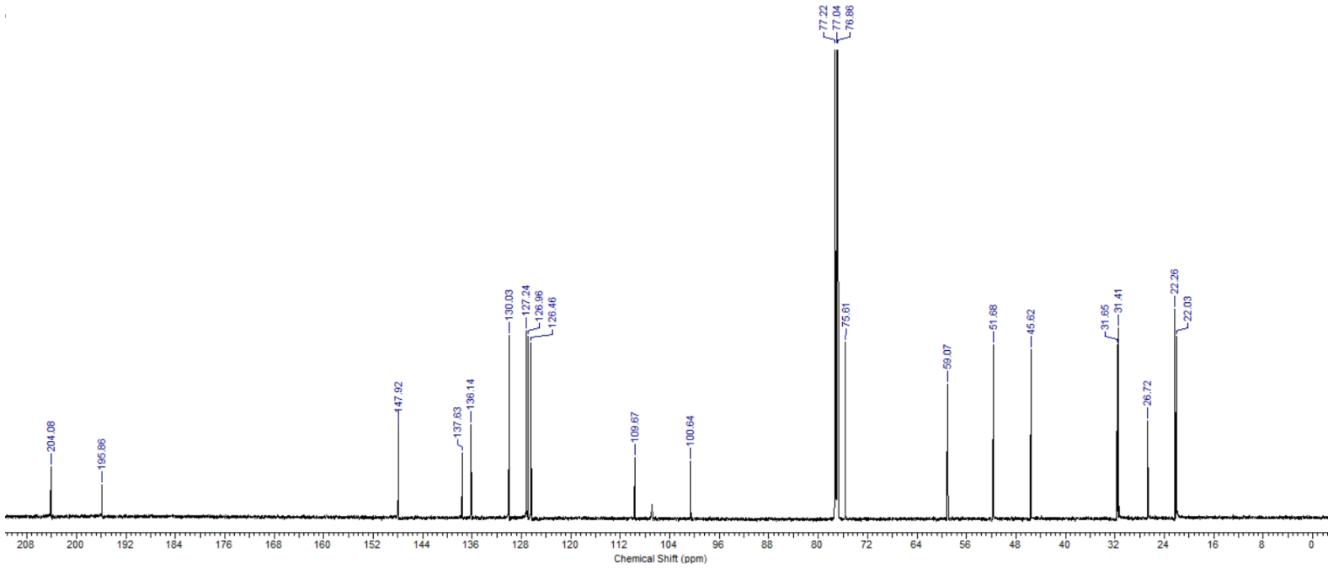

Figure S40: ^{13}C NMR spectrum of compound **3l**

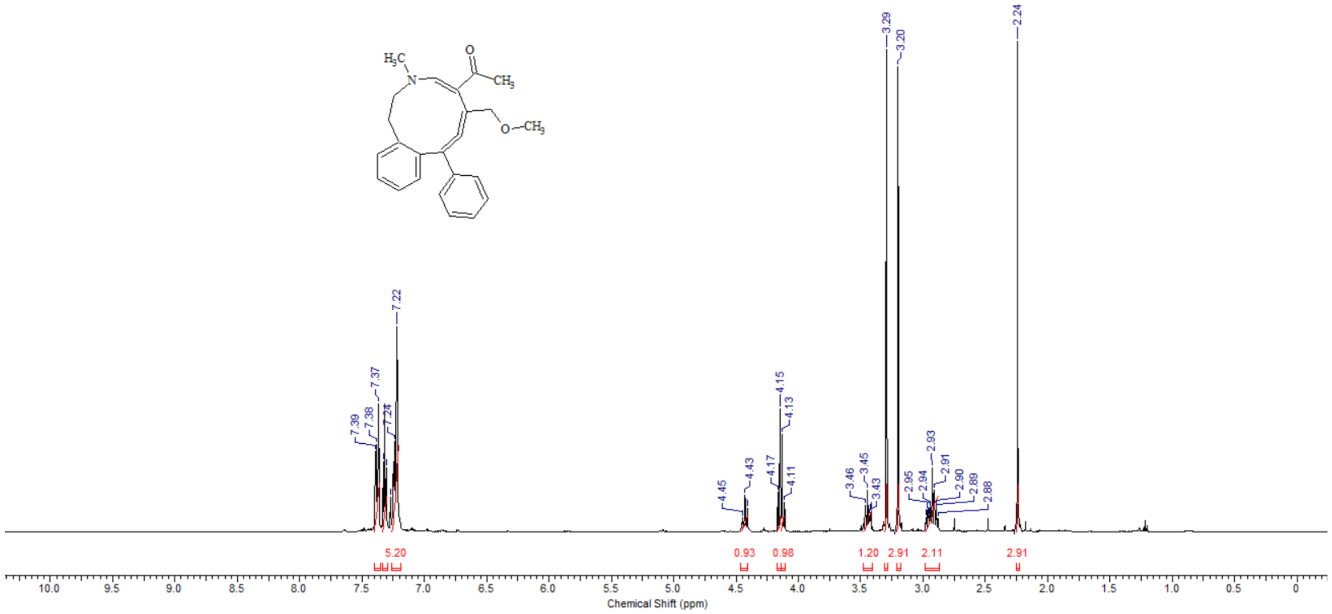

Figure S41: ^{1}H NMR spectrum of compound **3m**

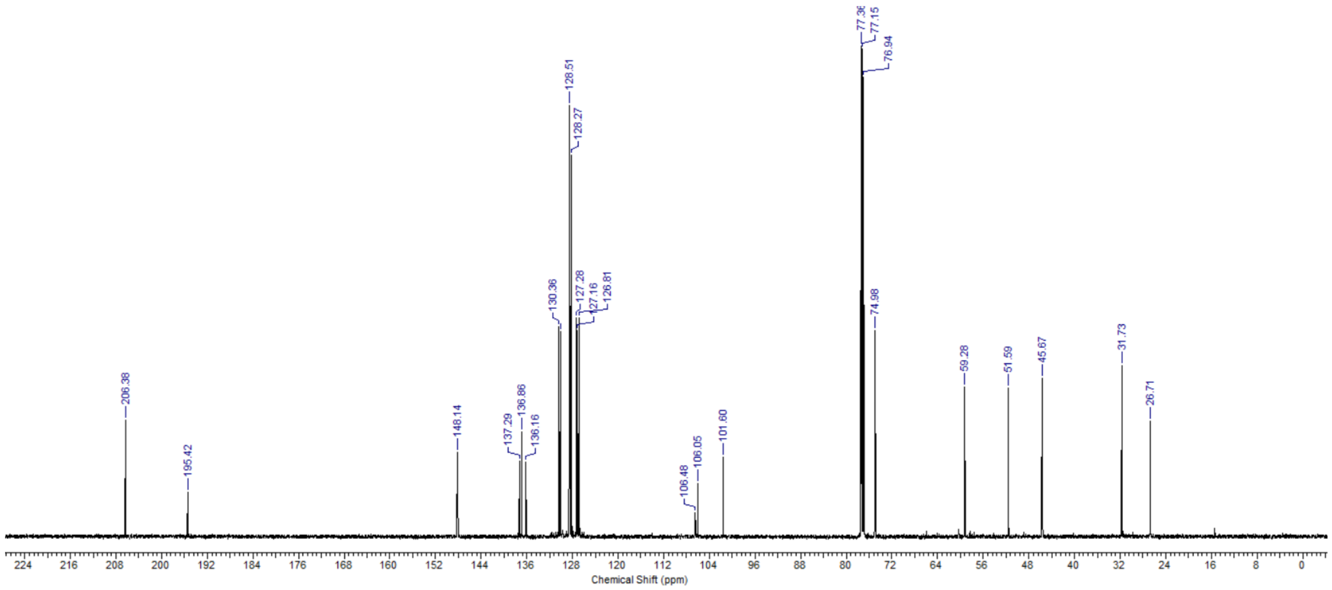

Figure S42: ^{13}C NMR spectrum of compound **3m**

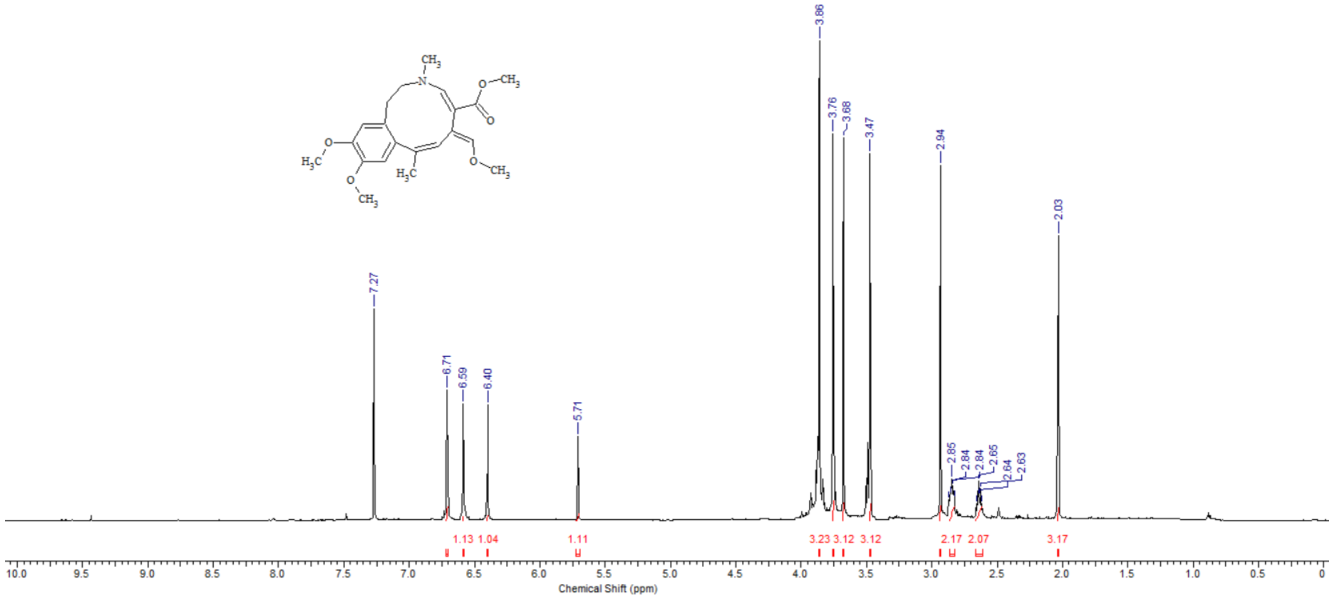

Figure S43: ^{1}H NMR spectrum of compound **4a**

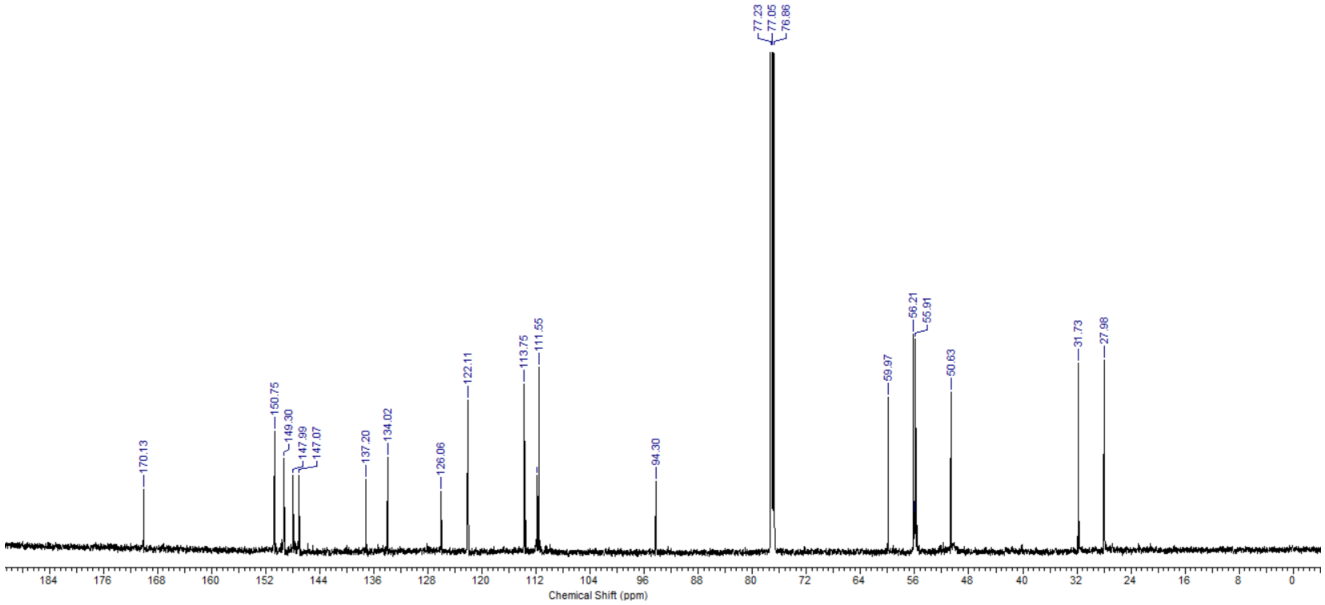

Figure S44: ^{13}C NMR spectrum of compound **4a**

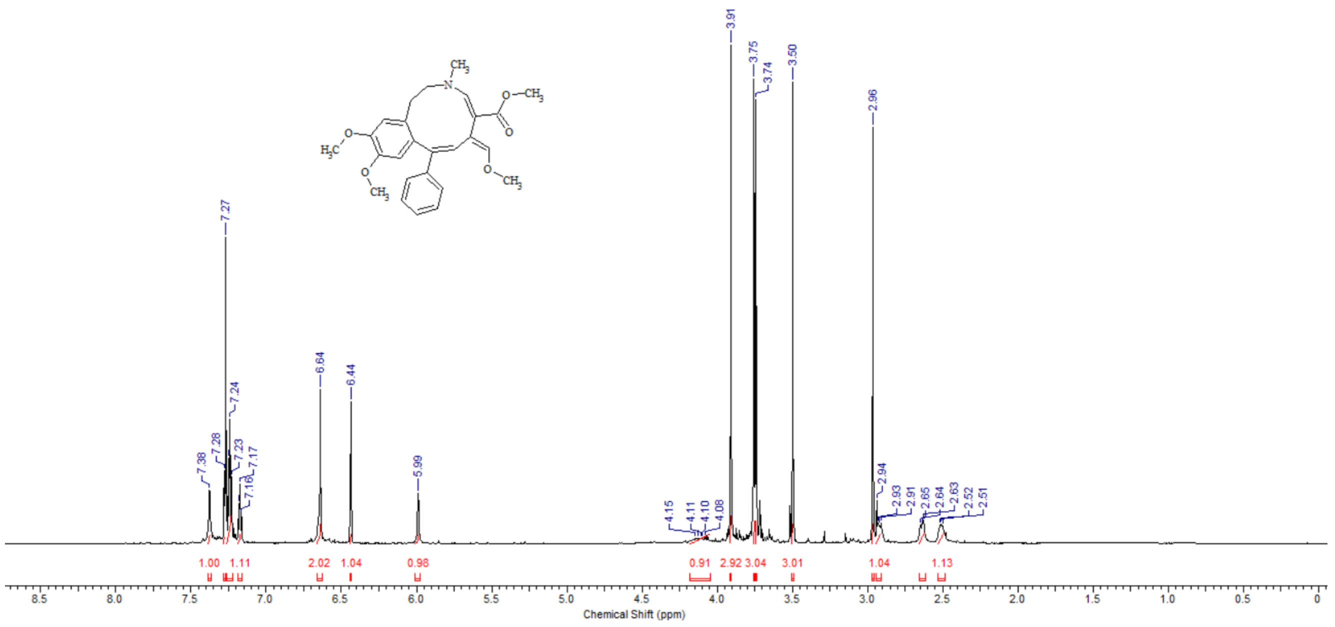

Figure S45: ^{1}H NMR spectrum of compound **4c**

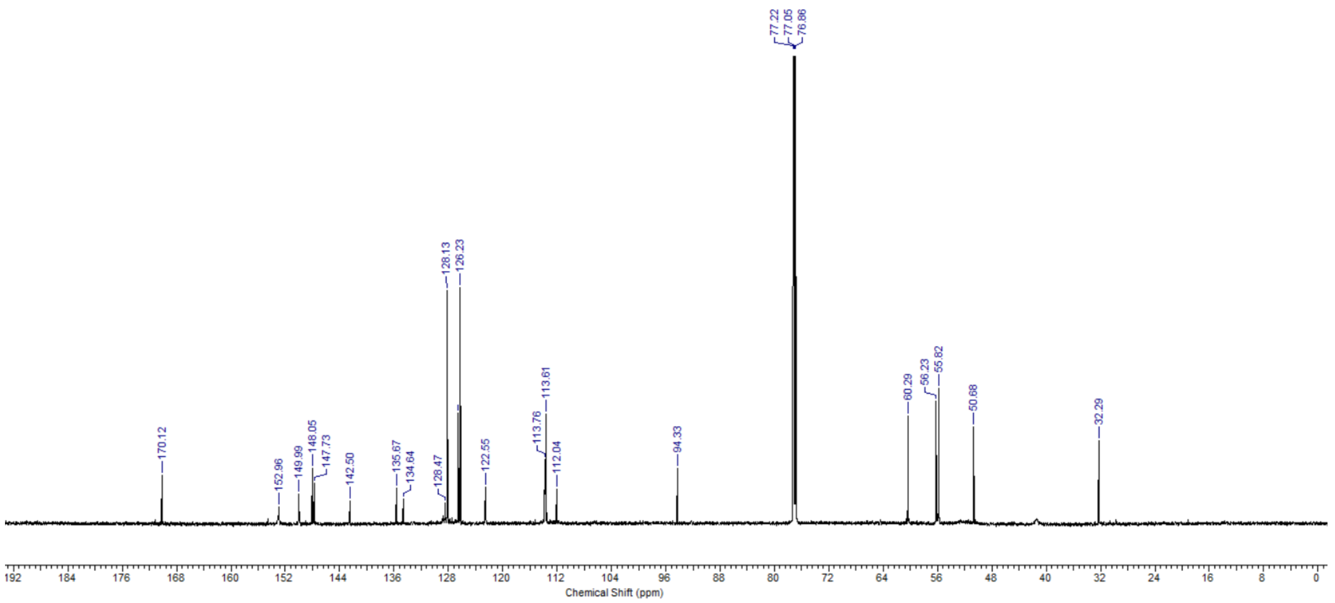

Figure S46: ^{13}C NMR spectrum of compound **4c**

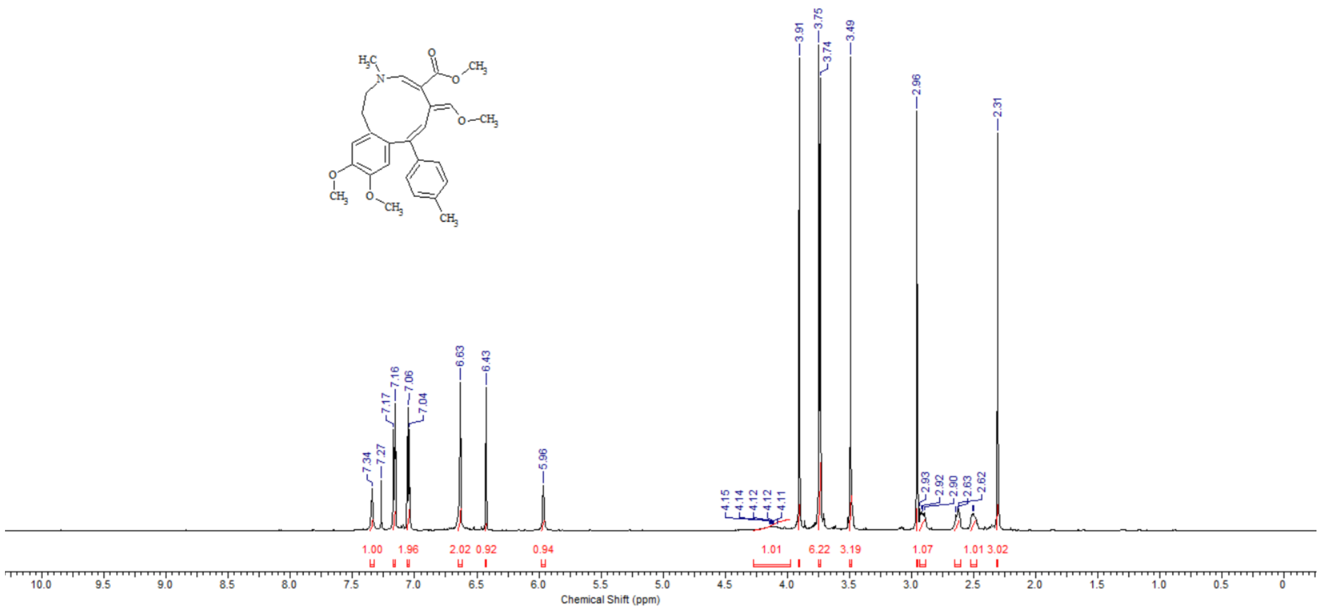

Figure S47: ^{1}H NMR spectrum of compound **4d**

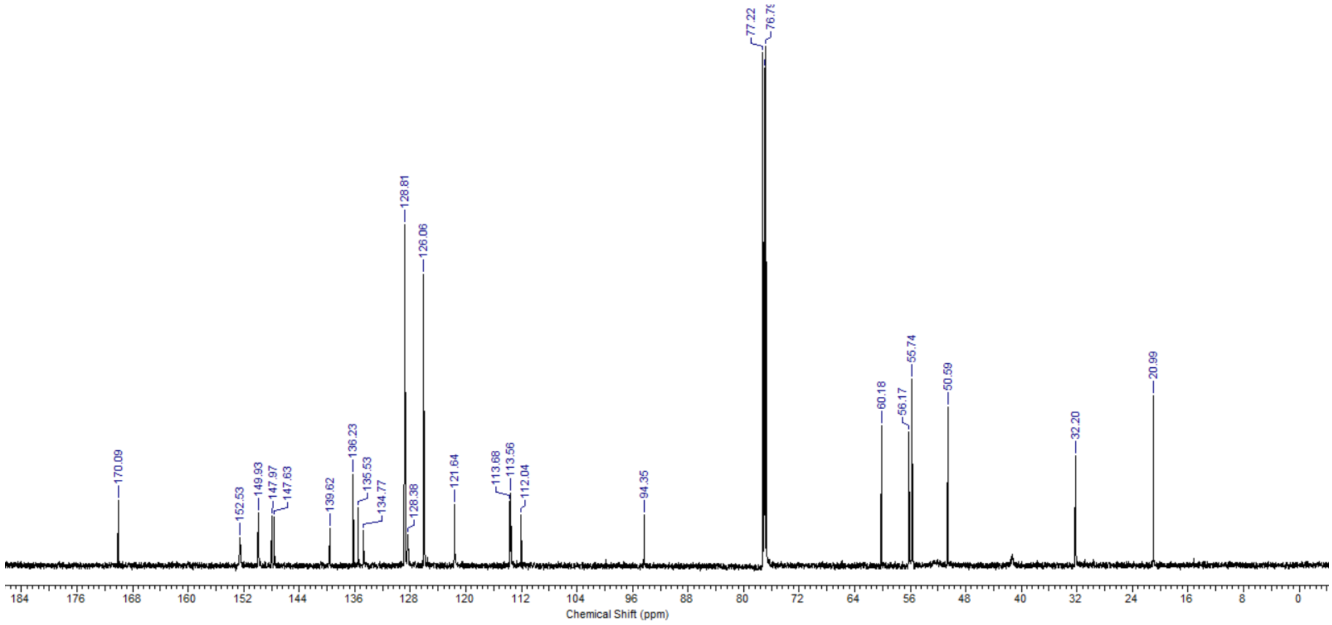

Figure S48: ^{13}C NMR spectrum of compound **4d**

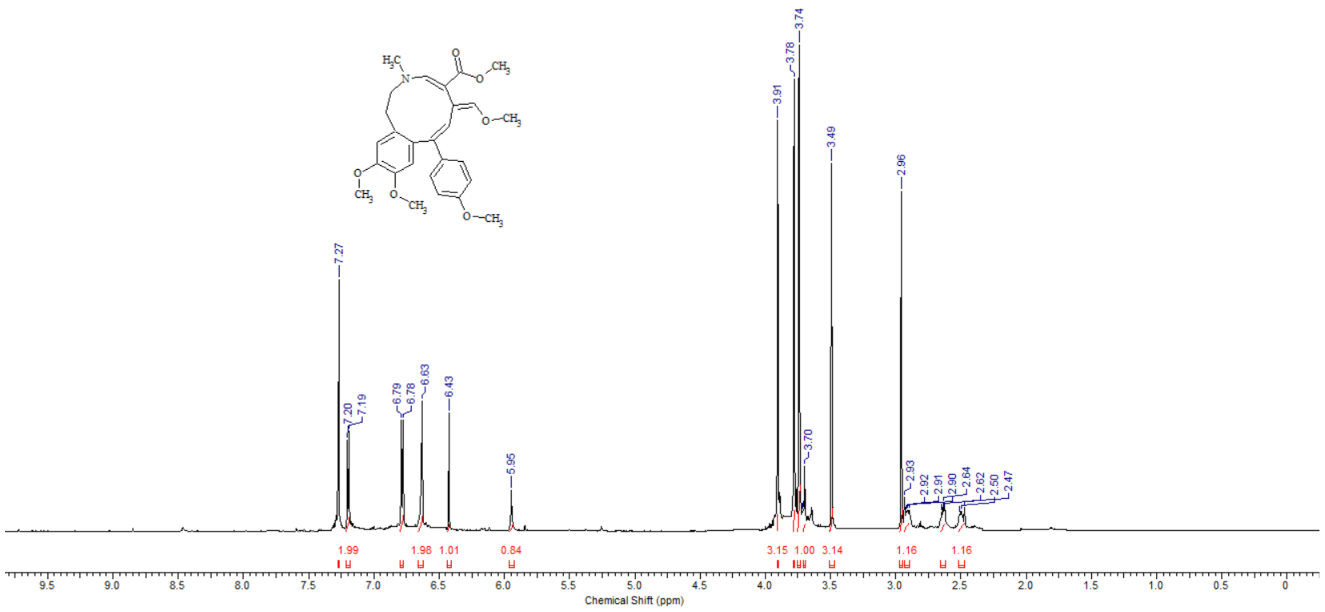

Figure S49: ^{1}H NMR spectrum of compound **4e**

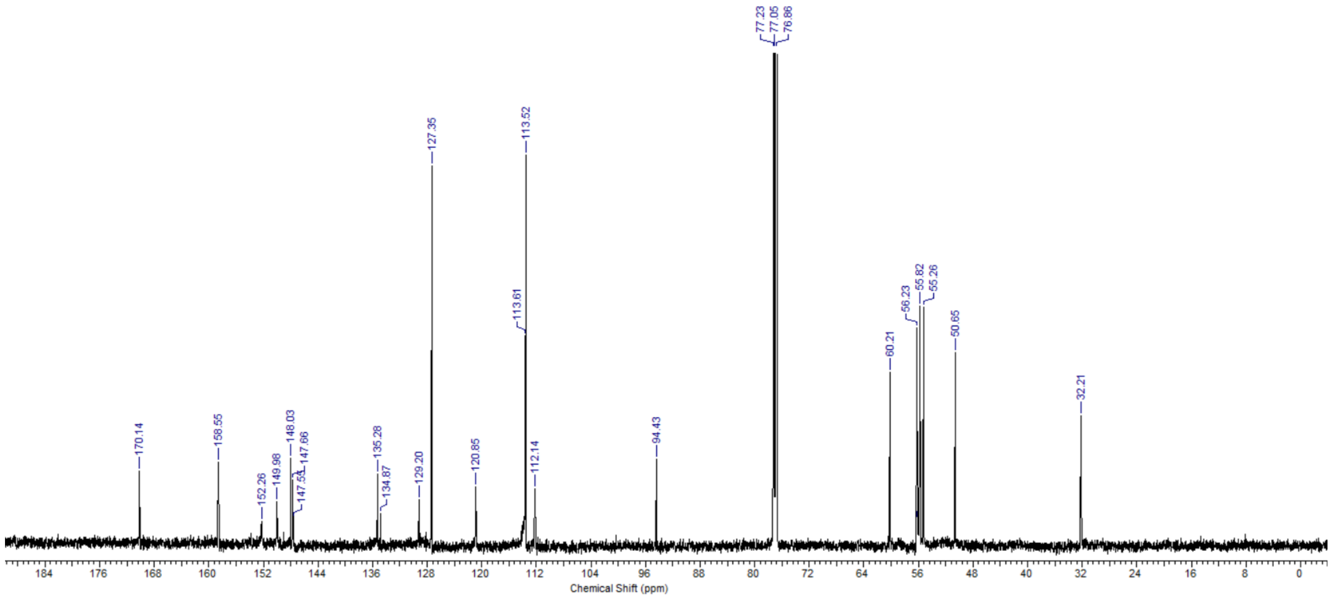

Figure S50: ^{13}C NMR spectrum of compound **4e**

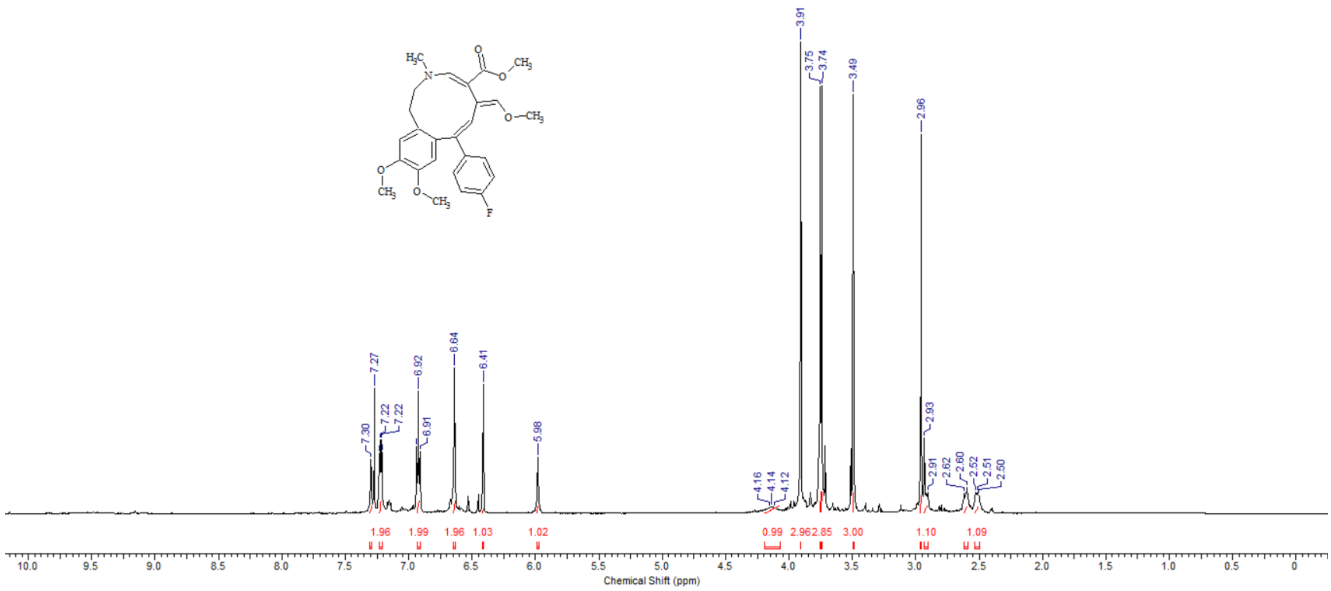

Figure S51: ^{1}H NMR spectrum of compound **4f**

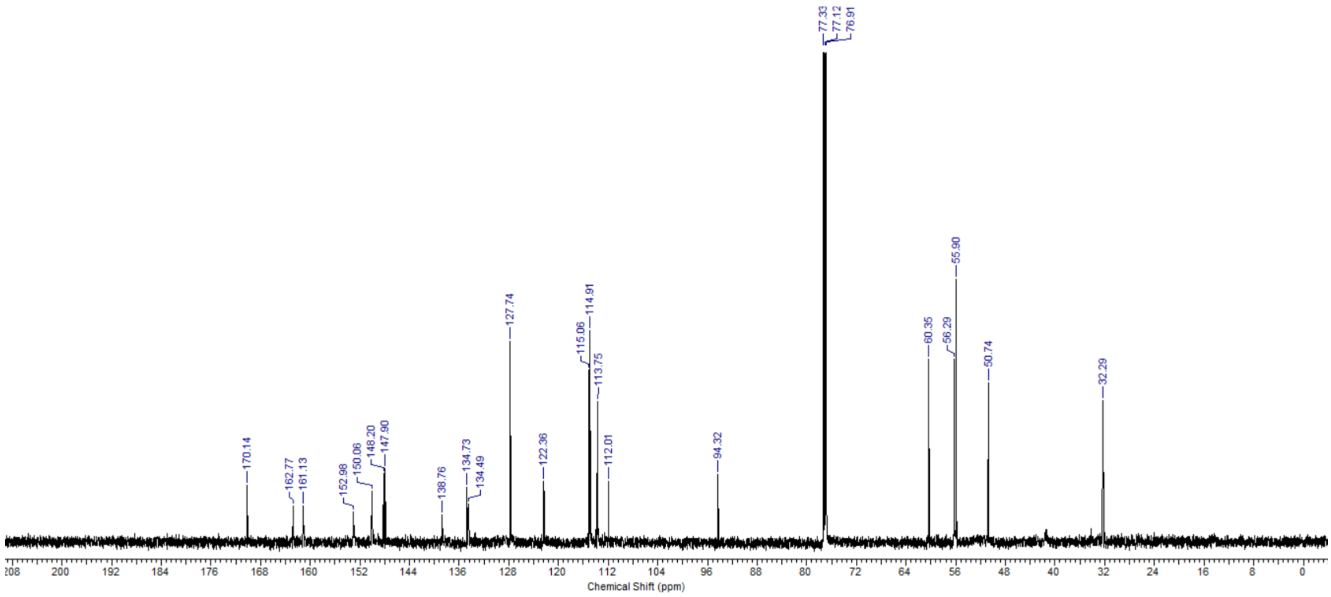

Figure S52: ^{13}C NMR spectrum of compound **4f**
